# Prominence of IL6, IGF, TLR, and Bioenergetics Pathway Perturbation in Lung Tissues of Scleroderma Patients With Pulmonary Fibrosis

**DOI:** 10.3389/fimmu.2020.00383

**Published:** 2020-03-10

**Authors:** Ludivine Renaud, Willian A. da Silveira, Naoko Takamura, Gary Hardiman, Carol Feghali-Bostwick

**Affiliations:** ^1^Department of Medicine, Medical University of South Carolina, Charleston, SC, United States; ^2^School of Biological Sciences, Institute for Global Food Security, Queens University Belfast, Belfast, United Kingdom

**Keywords:** scleroderma, systemic sclerosis, pulmonary fibrosis, idiopathic, microarray, interstitial lung disease

## Abstract

Scleroderma-associated pulmonary fibrosis (SSc-PF) and idiopathic pulmonary fibrosis (IPF) are two of many chronic fibroproliferative diseases that are responsible for nearly 45% of all deaths in developed countries. While sharing several pathobiological characteristics, they also have very distinct features. Currently no effective anti-fibrotic treatments exist that can halt the progression of PF or reverse it. Our goal is to uncover potential gene targets for the development of anti-fibrotic therapies efficacious in both diseases, and those specific to SSc-PF, by identifying universal pathways and molecules driving fibrosis in SSc-PF and IPF tissues as well as those unique to SSc-PF. Using DNA microarray data, a meta-analysis of the differentially expressed (DE) genes in SSc-PF and IPF lung tissues (diseased vs. normal) was performed followed by a full systems level analysis of the common and unique transcriptomic signatures obtained. Protein-protein interaction networks were generated to identify hub proteins and explore the data using the centrality principle. Our results suggest that therapeutic strategies targeting IL6 trans-signaling, *IGFBP2, IGFL2*, and the coagulation cascade may be efficacious in both SSc-PF and IPF. Further, our data suggest that the expression of matrikine-producing collagens is also perturbed in PF. Lastly, an overall perturbation of bioenergetics, specifically between glycolysis and fatty acid metabolism, was uncovered in SSc-PF. Our findings provide insights into potential targets for the development of anti-fibrotic therapies that could be effective in both IPF and SSc-PF.

## Introduction

Systemic sclerosis (SSc), commonly known as scleroderma, is a chronic and systemic autoimmune connective tissue disease characterized by proliferative/obliterative vasculopathy, immune dysregulation, and the development of fibrosis in the skin, lungs and other internal organs. SSc-associated pulmonary fibrosis (SSc-PF) is one of the leading causes of death in patients with SSc (1). SSc-PF is diffuse and displays inflammatory cell infiltration of the alveoli, interstitium and peribronchiolar tissues along with excessive proliferation of fibroblasts leading to extensive deposition of various extracellular matrix (ECM) proteins by mesenchymal cells, such as collagen type I and III, fibronectin and tenascin (2, 3). SSc is one of many chronic fibroproliferative diseases that are responsible for nearly 45% of all deaths in developed countries (4). No effective therapy currently exists that can halt the progression of fibrosis or reverse it.

SSc-PF shares pathobiologic characteristics with other lung diseases, especially with idiopathic pulmonary fibrosis (IPF), but also has distinct features (5). Results from clinical trials emphasized the differences in pathogenesis that exist between SSc-PF and IPF. Immunosuppressive therapies are more effective in SSc-PF than in IPF, i.e., cyclophosphamide, mycophenolate mofetil (MMF) (6, 7), and drugs targeting fibrotic pathways in PF have shown some benefits in IPF, i.e., nintedanib and pirfenidone (8, 9). Recently, nintedanib has also been shown to inhibit macrophage activation and ameliorate SSc-PF (10, 11). Treatment with an antagonist to lysophosphatidic acid receptor 1 (LPA1) showed promising results in a murine model of SSc-PF and in clinical trial of IPF patients by improving forced vital capacity (FVC) and reducing fibrosis and inflammation, even though the trial was terminated early (12, 13). Therapy with the anti-oxidant N-acetylcysteine (NAC) ameliorated pulmonary function in both SSc-PF and IPF patients (14, 15). Despite similar pathological features, the pursuit of a treatment that would be equally beneficial in IPF and SSc-PF has been challenging.

This study aims to characterize universal pathways and molecules driving fibrosis in SSc-PF and IPF lung tissues and identify potential gene targets for the development of anti-fibrotic therapies that could improve lung function in both diseases. In doing so, we will also identify the unique gene signature of SSc-PF and characterize therapeutic targets specific to SSc-PF. To achieve this goal, we performed a meta-analysis of the differentially expressed (DE) genes in SSc-PF and IPF lung tissues (diseased vs. normal) using microarray data, followed by a full systems level analysis of the common and unique transcriptomic signatures obtained. Gene, protein and metabolite interactions are key to decipher the biological meaning of systems (16). Using STRING (17), a database of known and predicted protein-protein interactions, we identified functionally important hub genes in shared and unique datasets for IPF and SSc-PF. Results from this analysis provide insights for the development of anti-fibrotic therapies that could be effective in both IPF and SSc-PF.

## Materials and Methods

### Study Population

Lung tissue samples were obtained from patients with SSc-PF (*n* = 13) and IPF (*n* = 13) who underwent lung transplantation at the University of Pittsburgh Medical Center, under a protocol approved by the Institutional Review Board. All patients with SSc met the American College of Rheumatology criteria for the diagnosis of SSc (18). Severe PF in SSc was defined as the presence of restrictive physiology, with a forced vital capacity (FVC) <55% of predicted. Patients with IPF were confirmed to have usual interstitial pneumonia (UIP) pathology without evidence of other known causes and no associated pulmonary arterial hypertension (PAH). Normal lung tissue specimens (*n* = 9) were obtained from organ donors whose lungs were not used for lung transplantation. Lung tissues were frozen prior to the extraction of total RNA.

### RNA Extraction and qRT-PCR Validation

Total RNA was extracted from frozen lung tissues using TRIzol (Thermo Fisher Scientific, USA) and purified using the RNeasy Kit (Qiagen, USA). RNA quality was determined by agarose gel electrophoresis as well as analysis of samples using an Agilent 2100 Bioanalyzer with an RNA integrity number ≥6. For cDNA synthesis, 1,000 ng of total RNA was used with these reagents (Invitrogen, USA): Oligo(dT)12-18 Primer (Catalog # 18418012) and SuperScript™ IV (Catalog # 18090010). For qRT-PCR, the TaqMan™ Gene Expression Master Mix (Applied Biosystems, USA, Catalog # 4369016) was used along with the following primers: *IGFBP2* (Hs01040719_m1); *IGFL2* (Hs01389017_m1); *IL-6* (Hs00985639_m1); *TLR8* (Hs00152972_m1); *B2M* as housekeeping gene (Hs00187842_m1) on a StepOnePlus real time PCR system (Applied Biosystems, USA). The sample size for qRT-PCR validation was *n* = 8 for NL and SSc-PF, and *n* = 12 for IPF. For statistical analysis, a Kruskal-Wallis test was performed followed by Dunn's multiple comparisons test with significance set at *p* < 0.05. Error bars indicate standard error of the mean (SEM).

### Gene Level Analysis: Microarray

Gene expression profiling was performed by microarray analysis using HumanRef-8 v3.0 BeadChips (Illumina, USA) containing 25,440 annotated genes. After sample hybridization, BeadChips were scanned using an Illumina BeadChip Array Reader. Intensity data was loaded in Limma version 3.40.2 in R version 3.6.0 and normalization between arrays was performed using the quantile method. Normalized expression was log2 transformed before being fitted to a linear model and differential expression analysis was performed for the following comparisons: SSc-PF vs. normal (NL) and IPF vs. NL. For each gene, Limma reported the estimated log2 fold change (log2FC) and provided a false discovery rate (FDR) adjusted q-value. FDR is the expected fraction of false positive tests among significant tests and was calculated using the Benjamini-Hochberg multiple testing adjustment procedure. Differentially expressed (DE) genes were identified based on the following criteria: *q* < 0.1, log2FC > 1: upregulated (equivalent to linear FC increase of 2), log2FC < −1: downregulated (equivalent to linear FC decrease of 2). The dataset is deposited on the Gene Expression Omnibus database with GSE48149 accession number (https://www.ncbi.nlm.nih.gov/geo/). The Genotype-Tissue Expression (GTEx) Portal (https://gtexportal.org/home/) was accessed on 05/01/2019 to obtain information on IGFBP2 and IGFL2 genes.

Hierarchical clustering was generated using MORPHEUS, a versatile matrix visualization and analysis software (https://software.broadinstitute.org/morpheus). The normalized data for all 425 DE genes (common and unique to both diseases) was uploaded to the site and the parameters hierarchical clustering, one minus cosine similarity based, linkage method on average and cluster on columns were selected (relative color scheme 0.63).

### Systems Level Analysis

#### Functional Enrichment and Gene Ontology (GO)

Functional enrichment was performed on DE genes (*q* < 0.1, linear fold change of 2) using ToppFun (19) (ToppGene Suite), a portal for gene list enrichment analysis and candidate gene prioritization based on functional annotations and protein interactions network. ToppFun specifically extracts information from transcriptome, gene ontology (GO) and biological pathway annotations. GO terms were then visualized using REViGO (20), a tool that summarizes long lists of GO terms by removing redundant ones. The remaining terms were visualized in semantic similarity-based scatterplots and treemaps, a two-level hierarchy of GO terms that grouped cluster representatives under “superclusters” of related terms.

#### Protein-Protein Interaction Network (STRING)

The Search Tool for the Retrieval Of Interacting Genes/Proteins (STRING) is a database purposely designed to gather information on protein-protein interactions (direct experimental evidence and *de novo* predictions via computational approaches) and protein associations in the context of metabolic, signaling or transcriptional pathways (17). DE genes were entered (*q* < 0.1, linear FC of 2) using the following settings: lines thickness indicates the strength of data support (high confidence 0.7), the active interaction sources selected were textmining, experiments, databases, coexpression and gene fusion, number of interactors in the 1st shell were limited to query proteins only and in the 2nd shell were limited to 2 interactors; disconnected nodes were hidden in the network. In this study, hub genes are defined as genes with at least 5 links (connections).

#### Pathway Impact Analyses and Coherent Cascades

Pathway impact analysis was performed using iPathwayGuide (Advaita) on DE genes (*q* < 0.1, linear fold change of 1.5 to capture biologically significant perturbations of relevant pathways) to include important biological factors such as the magnitude of the expression change of each gene, the position of the DE genes in the given pathways, the topology of the pathway that describes how these genes interact, and the type of signaling interactions between them (21). This approach better captures the enrichment and perturbation of a given pathway and applies coherent cascade analysis to identify putative mechanisms. The impact analysis is modeled on KEGG Pathways.

## Results

### Differential Expression Analysis

**Figure 1:**
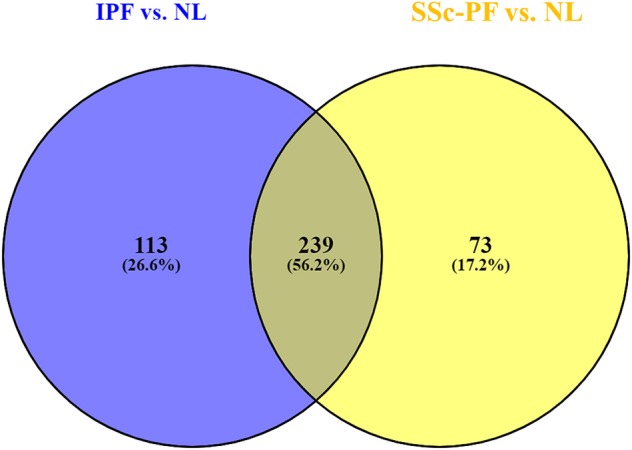
Meta-analysis of DE genes in IPF and SSc-PF. Differential expression analysis revealed 312 DE genes in the SSc-PF vs. NL comparison, and 352 DE genes in the IPF vs. NL (*q* < 0.1, log2FC > |1|); 239 DE genes were commonly deregulated in both diseases (intersection), representing 56.2% of all DE genes (intersection and unique lists combined = 425). Conversely, 113 and 73 DE genes were exclusively perturbed in IPF and SSc-PF, respectively.

Differential expression analysis revealed 312 DE genes in the SSc-PF vs. NL comparison, and 352 DE genes in the IPF vs. NL comparison (*q* < 0.1, log2FC > |1|, Supplementary Tables 1, 2). Out of 425 DE genes identified (common and unique lists combined), 239 DE genes were commonly deregulated in both diseases (Figure 1, intersection), representing 56.2% of all DE genes. Conversely, 113 and 73 DE genes were exclusively perturbed in IPF and SSc-PF, respectively.

**Figure 2:**
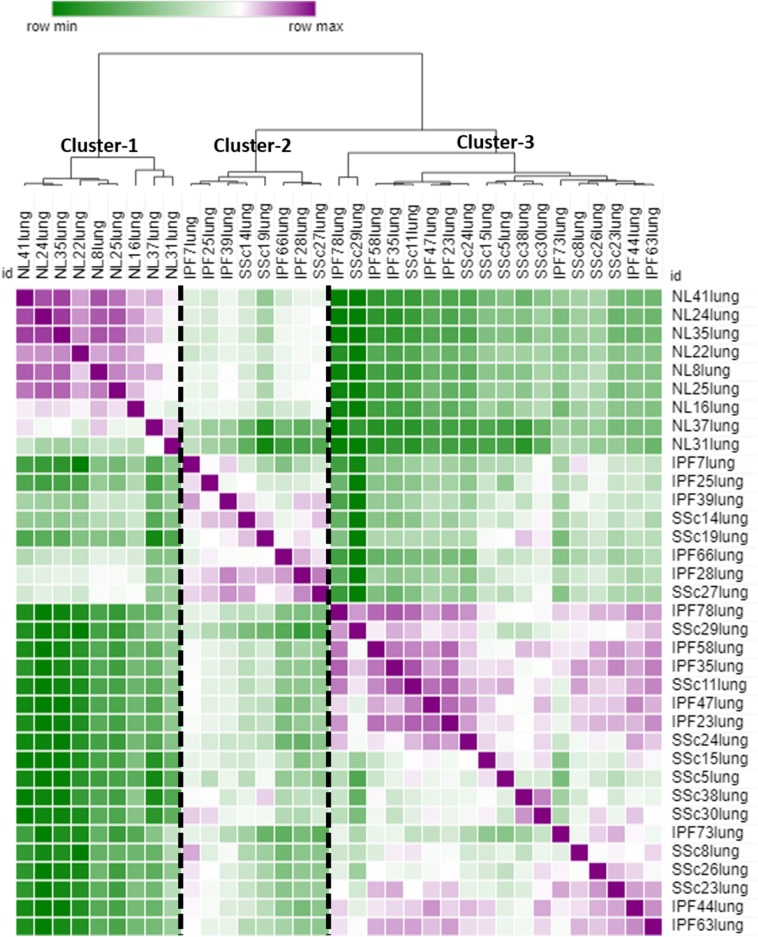
Similarity matrix. The raw data for all 425 DE genes (common and unique to both diseases) was uploaded to MORPHEUS to generate a similarity matrix. Three main clusters are apparent, cluster-1 made up of NL samples only, cluster-2 and cluster-3 made up of SSc-PF and IPF samples intertwined, emphasizing that the gene signatures of these 2 diseases are very similar.

Hierarchical clustering of all 425 DE genes showed 3 main clusters: cluster-1 contains all NL samples, cluster-2 and cluster-3 comprise IPF and SSc-PF samples intertwined together (Figure 2), emphasizing that IPF and SSc-PF patients have a similar transcriptomic perturbation. Heatmaps of common and unique DE genes were also generated (Supplementary Figures 1–3).

#### Upregulation of IGFBP2 and IGFL2 Is Common to Both Diseases

We and others have previously shown that the insulin-like growth factors (IGFs) and their binding proteins (IGFBPs) are key players in the development and progression of pulmonary fibrosis (22–27). *IGFBP3, IGFBP5*, and *IGFBP7* have been previously shown to be upregulated in both lung tissues and lung fibroblasts from patients with SSc-PF and IPF (24–26). *IGFBP4* is downregulated in SSc lung fibroblasts (27), and upregulated in IPF lung tissues (28, 29). Here we describe perturbation of the IGF signaling system in SSc-PF and IPF, specifically upregulation of *IGFBP2* and *IGF-like family member 2* (*IGFL2*) (Table 1). At homeostasis, *IGFBP2* is detectable in several organs and without obvious gender differences in fibroblasts, lung and skin, while *IGFL2* is highly expressed in healthy skin samples of both male and female donors (Supplementary Figures 4, 5).

**Table 1:** Genes deregulated in the IGF pathway.

**SSc-PF vs. NL**				**IPF vs. NL**			
**Symbol**	**Entrez_ID**	**log2FC**	***q*-value**	**Symbol**	**Entrez_ID**	**log2FC**	***q*-value**
*IGFBP2*	3485	1.50	2.16E-05	*IGFBP2*	3485	1.27	2.30E-04
*IGFBP4*	3487	0.95	8.37E-04	*IGFBP4*	3487	0.90	1.58E-03
*IGFBP7*	3490	0.63	2.24E-03	*IGFL2*	147920	1.17	4.04E-03
*IGFL2*	147920	1.07	7.97E-03	*IGFBP7*	3490	0.56	6.55E-03
*IGFL1*	374918	0.39	2.72E-02	*IGFBP5*	3488	0.77	4.70E-02
*IGFBP5*	3488	0.78	4.24E-02	*IGFL1*	374918	0.31	8.34E-02
*IGF1*	3479	0.42	1.07E-01	*IGF2BP3*	10643	−0.26	1.09E-01
				*IGF1*	3479	0.41	1.22E-01

*DE upregulated genes are highlighted in red (q < 0.1, log2FC > |1|, representing a 2 linear fold change increase in gene expression). Genes are sorted by q-value from smallest to largest*.

#### Perturbation of Matrikine-Producing Collagens in SSc-PF and IPF

Collagen-derived matrikines are peptides generated by partial proteolysis of certain collagens that can regulate cell activity (30). Out of the 9 DE collagen genes identified in the intersection of SSc-PF and IPF (Table 2), 4 have been identified as sources of matrikines: *COL1A1, COL1A2, COL15A1*, and *COL17A1* (30, 31). Note that all 4 collagens are upregulated in both diseases by at least a linear fold change increase of 2.

**Table 2:** Perturbation of matrikine-producing collagen genes.

**SSc-PF vs. NL**				**IPF vs. NL**			
**Symbol**	**Entrez_ID**	**log2FC**	***q*****-value**	**Symbol**	**Entrez_ID**	**log2FC**	***q*****-value**
*COL9A2*	1298	1.12	1.91E-05	*COL9A2*	1298	1.10	2.60E-05
*COL7A1*	1294	2.14	3.20E-05	COL1A1*	1277	2.29	4.71E-05
COL15A1*	1306	1.96	5.14E-05	COL17A1*	1308	1.94	1.31E-04
COL17A1*	1308	2.06	5.21E-05	*COL7A1*	1294	1.92	1.56E-04
COL1A1*	1277	2.09	1.42E-04	COL15A1*	1306	1.59	6.54E-04
*COL10A1*	1300	1.31	5.53E-04	COL1A2*	1278	1.69	1.71E-03
COL1A2*	1278	1.68	1.63E-03	*COL10A1*	1300	1.18	1.79E-03
*COL3A1*	1281	1.84	5.43E-03	*COL3A1*	1281	1.83	6.12E-03
*COL5A2*	1290	1.13	1.46E-02	*COL5A2*	1290	1.08	1.96E-02

DE upregulated genes are highlighted in red (q ≤ 0.1, log2FC ≥ 1 representing a linear fold change of 2). Genes are sorted by q-value from smallest to largest. Bold^*^matrikine-producing collagen genes.

### Systems Level Analysis

Our systems level analysis examined gene ontology and pathway impact analysis to try to decipher the biological relevance of the gene perturbation observed by taking into consideration the position and role of every gene in the pathway, the direction and type of signal from one gene to another, and feedback mechanisms that may exist (21). The meta-analysis revealed 56 enriched pathways in the IPF vs. NL comparison, and 67 in the SSc-PF vs. NL. A total of 48 pathways were commonly enriched in both diseases (intersection), while 8 and 19 pathways were exclusively perturbed in IPF and SSc-PF, respectively (Supplementary Figure 6).

We also analyzed STRING-generated networks for protein-protein interactions to identify hub genes in the intersection and unique gene sets using the principle of “network centrality” which states that genes with the highest degree of centrality (more connected) are three times more likely to be essential than genes with a smaller number of connection to other genes (32, 33).

#### Intersection

**Figure 3:**
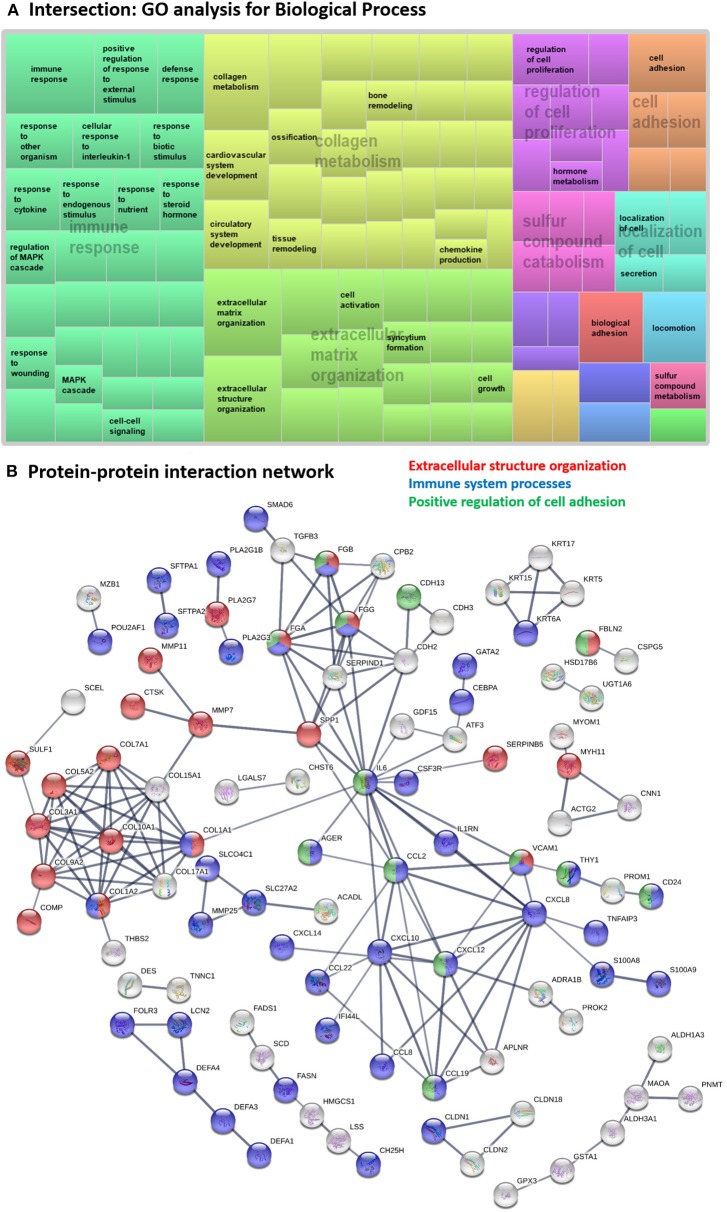
Systems level analysis—intersection. **(A)** Gene ontology analysis for biological process. Data obtained from ToppFun and visualized with REViGO treemaps (abs_log10_pvalue). **(B)** Protein-protein interaction network generated by STRING.

The meta-analysis revealed 239 DE genes at the intersection of IPF and SSc-PF. GO analysis of these genes revealed perturbation of “immune response,” “extracellular matrix organization,” and “collagen metabolism” biological processes as well as enrichment of “ECM-receptor interaction,” “cytokine-cytokine receptor interaction,” “chemokine,” and “IL-17” signaling pathways (Figure 3A and Supplementary Figures 6, 7), reflecting ongoing inflammatory immune responses in both IPF and SSc-PF, even at such a late stage of pulmonary fibrosis.

Hub proteins identified in the functional protein association network generated using STRING (Figure 3B) included interleukin 6 (IL6), several CXC chemokine family members (CXCL8, CXCL10, CXCL12), two CC motif chemokine ligands (CCL2, CCL19), APLNR, SERPIND1, VCAM1, several coagulation factor fibrinogens (FGA, FGB, FGG), CDH2, SPP1 and several collagens proteins (COL1A1, COL1A2, COL3A1, COL5A2, COL7A1, COL9A2, COL10A1, COL15A1, COL17A1). Note that CXCL8, APLNR, FGA, and FGB were not DE genes but were hub proteins in the network as 2nd shell interactors. All identified hub proteins in this network were upregulated genes, except *FGG* which was downregulated (Supplementary Table 3) in both DE analyses, showing that these genes are similarly regulated in both diseases.

The fibrinogens and VCAM1 are involved in all 3 major biological processes “extracellular structure organization,” “immune system processes,” and “positive regulation of cell adhesion,” while IL6, CCL2, CCL19, and CXCL12 are involved in both “immune system processes” and “positive regulation of cell adhesion” terms. COL1A1 and COL1A2 are hit genes in “extracellular structure organization” and “immune system processes” GO terms.

#### Unique to SSc-PF

**Figure 4:**
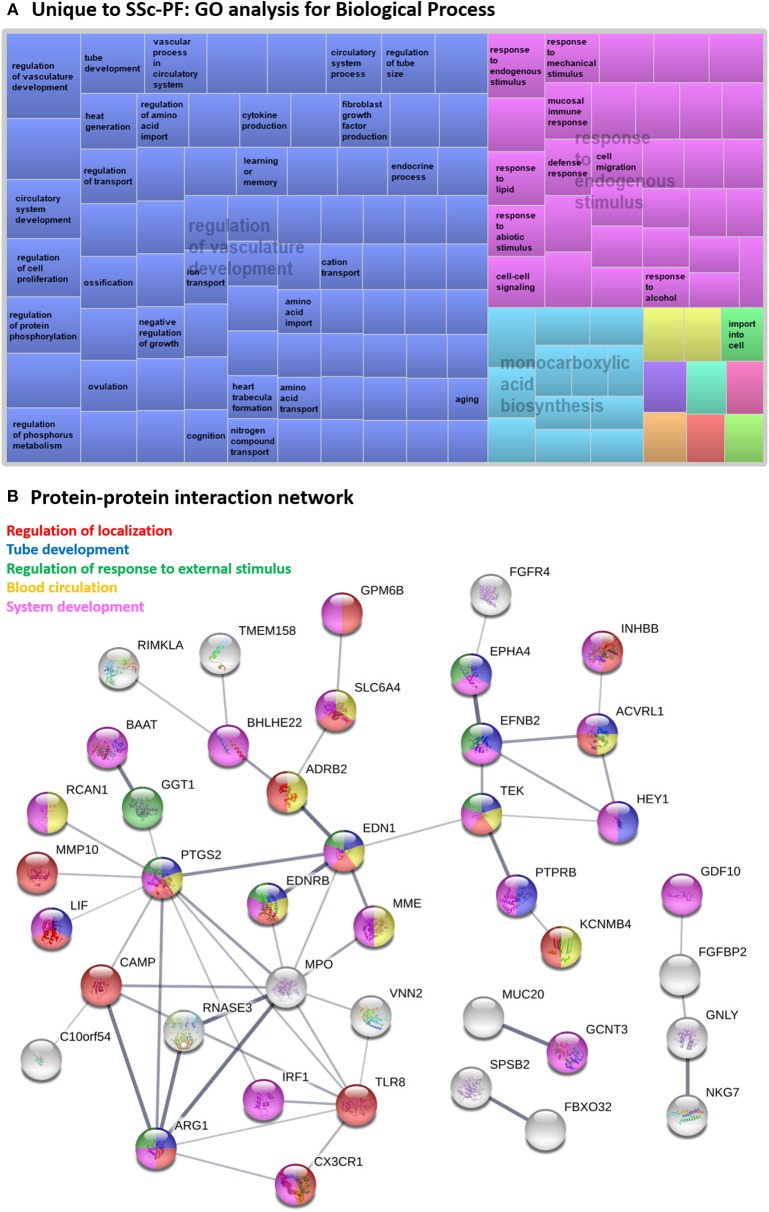
Systems level analysis—unique to SSc-PF. **(A)** Gene ontology analysis for biological process. Data obtained from ToppFun and visualized with REViGO treemaps (abs_log10_pvalue). **(B)** Protein-protein interaction network generated by STRING.

**Figure 5:**
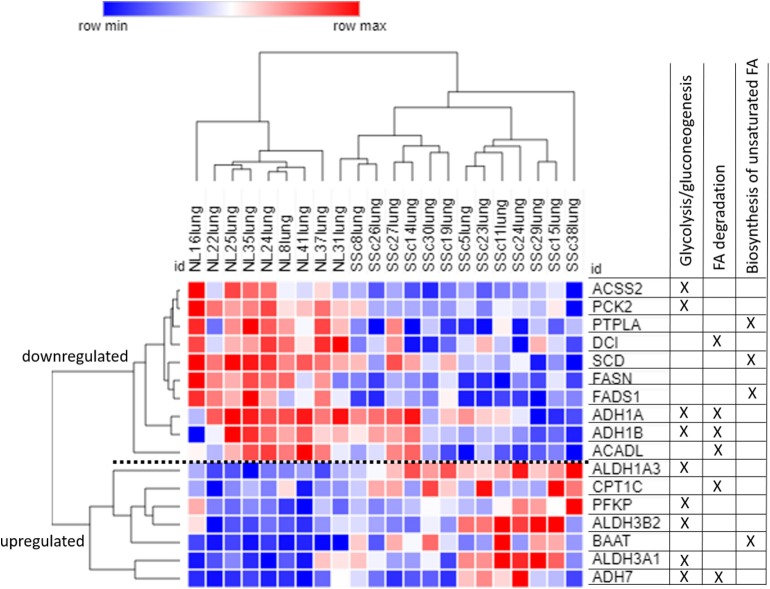
Heatmap of DE genes pertaining to fatty acid metabolism and glycolysis. The clustering is established based on one minus cosine similarity on both columns and rows. Table on the right side shows in which pathways each gene is involved, note that some genes are associated with more than one pathway. Blue: downregulation, red: upregulation.

The 73 DE genes exclusive to SSc-PF mainly pertained to “regulation of vasculature development” and “response to endogenous stimulus” biological processes as these terms were super clusters in the GO analysis (Figure 4A and Supplementary Figure 7). Pathways related to immunity and inflammation were also enriched, including “C-type lectin receptor,” “TNF,” and “PI3K-Akt” signaling pathways, suggesting that both innate and adaptive immune responses are still active in SSc-PF even in late stage disease (Supplementary Figure 6). Additionally, pathways related to metabolism and bioenergetics were also perturbed in SSc-PF, including “fatty acid (FA) degradation,” “biosynthesis of unsaturated FA,” and “glycolysis/gluconeogenesis” (Table 3). Figure 5 is a heatmap showing the DE genes associated with these pathways and how they cluster. For column clustering, all NL and all SSc-PF samples clustered together except for NL31 that clustered with SSc-PF samples. For row clustering, several of the DE genes involved were downregulated in SSc-PF as compared to NL (*ACSS2, PCK2, PTPLA, DCI, SCD, FASN, FADS1, ADH1A, ADH1B*, and *ACADL*). On the other end, *ALDH1A3, ALDH3A1, ALDH3B2, CPT1C, PFKP, BAAT*, and *ADH7* were upregulated in SSc-PF. These findings reflect an overall deregulation of FA metabolism and glycolysis in SSc-PF patients.

**Table 3:** Enriched pathways in SSc-PF pertaining to FA metabolism and glycolysis.

**Pathway**	**SSc-PF vs. NL** **(*p*-value)**
Fatty acid degradation	0.044
Biosynthesis of unsaturated fatty acids	0.022
Glycolysis/gluconeogenesis	0.006

*p < 0.05*.

Several hub proteins were evident in the STRING network generated (Figure 4B), including toll-like receptor 8 (TLR8), myeloperoxidase (MPO), prostaglandin-endoperoxide synthase 2 (PTGS2 aka COX2), arginase 1 (ARG1), and endothelin 1 (EDN1) genes. PTGS2, ARG1, and EDN1 are associated with several enriched GO terms: “regulation of localization,” “tube development,” “regulation of response to external stimulus,” and “system development.” PTGS2 and EDN1 are also present in “blood circulation” process. All identified hub proteins in this network were downregulated genes, except *PTGS2* which was upregulated (Supplementary Table 4). Note that EDN1 was not a DE gene but was a hub gene in the network as a 2nd shell interactor.

#### Unique to IPF

**Figure 6:**
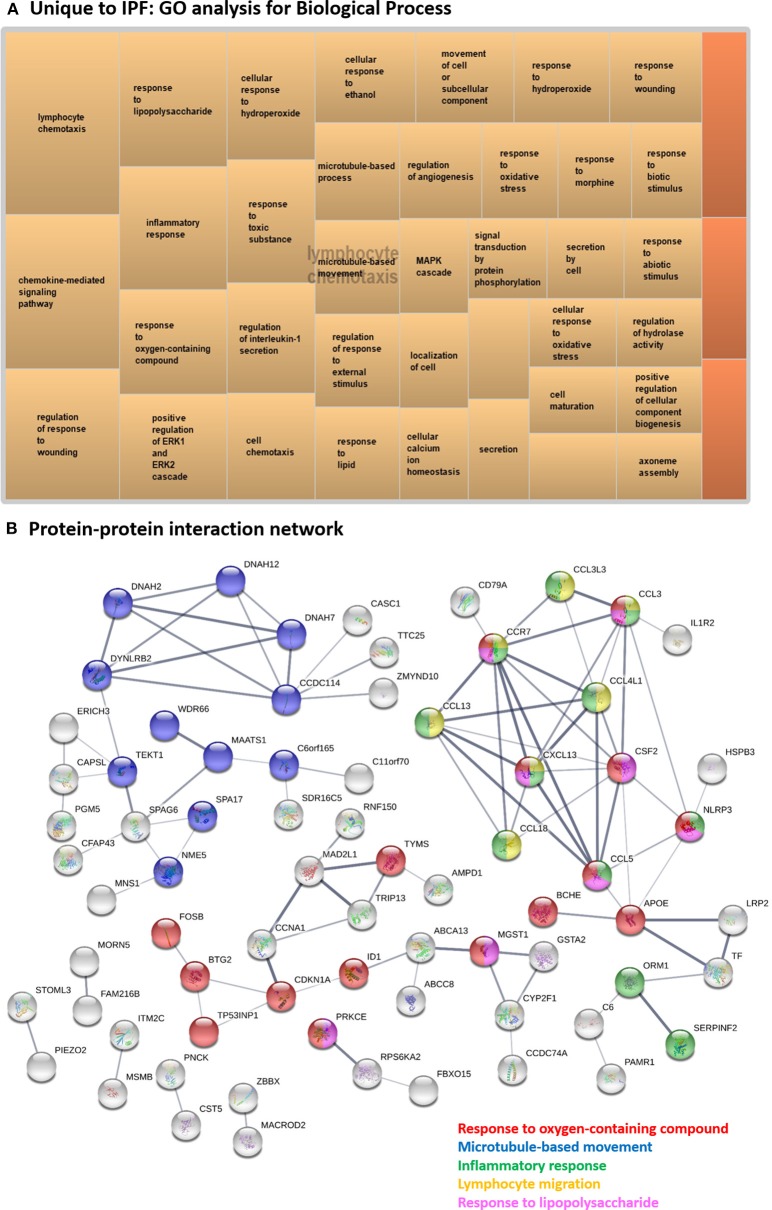
Systems level analysis—unique to IPF. **(A)** Gene ontology analysis for biological process. Data obtained from ToppFun and visualized with REViGO treemaps (abs_log10_pvalue). **(B)** Protein-protein interaction network generated by STRING.

One hundred and thirteen DE genes were unique to IPF and these generated the super-cluster “lymphocyte chemotaxis” in the GO analysis (Figure 6A and Supplementary Figure 7), emphasizing the importance of the immune response to external stimuli in IPF. The immune signature was also present in the unique pathways to SSc-PF, such as “intestinal immune network for IgA production,” “toll-like receptor signaling pathway,” emphasizing on-going innate immune response (Supplementary Figure 6).

Several hub proteins are associated with multiple enriched GO terms, including “response to oxygen-containing compound,” “microtubule-based movement,” “inflammatory response,” “lymphocyte migration,” and “response to lipopolysaccharide”: CSF2, CCR7, CCL3, CCL4L1, CCL13, CXCL13, CCL5, and NLRP3 (Figure 6B). APOE and CCDC114 were hub proteins only associated with “response to oxygen-containing compound” and “microtubule-based movement,” respectively. All identified hub proteins in this network were upregulated DE genes, except *CSF2* which was downregulated (Supplementary Table 5). Note that CCDC114 was not a DE gene but was a hub gene in the network as a 2nd shell interactor.

### Validation of IGFBP2, IGFL2, IL6, and TLR8 by qRT-PCR

**Figure 7:**
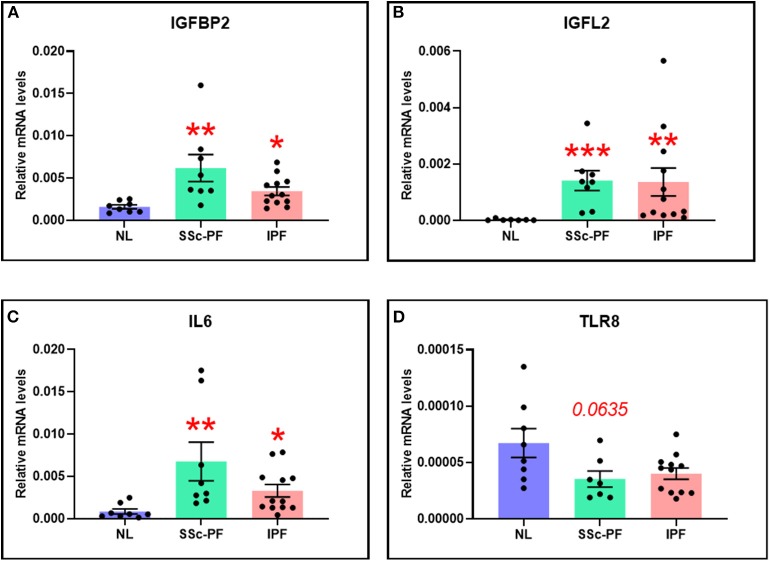
The expression levels of **(A)** IGFBP2, **(B)** IGFL2, **(C)** IL6, and **(D)** TLR8 were quantified in tissue lysates from SSc-PF (*n* = 8) and IPF (*n* = 12) patients and healthy individuals (NL, *n* = 8). *p < 0.05, **p < 0.01, ***p < 0.001 vs. NL. Error bars = SEM.

The DE and systems level analyses emphasized the deregulation and importance of *IGFBP2, IGFL2*, and *IL6* in both diseases, as well as *TLR8* in SSc-PF. Using qRT-PCR, these genes of interest were validated (Figure 7). In both SSc-PF and IPF lung samples, the expression levels of *IGFBP2, IGFL2*, and *IL6* were significantly increased compared to NL (Figures 7A–C), and *TLR8* was noticeably decreased in SSc-PF lungs, albeit not significantly (Figure 7D). These results are consistent with and validate the microarray data.

### Comparing Inflammatory Responses in IPF and SSc-PF

**Figure 8:**
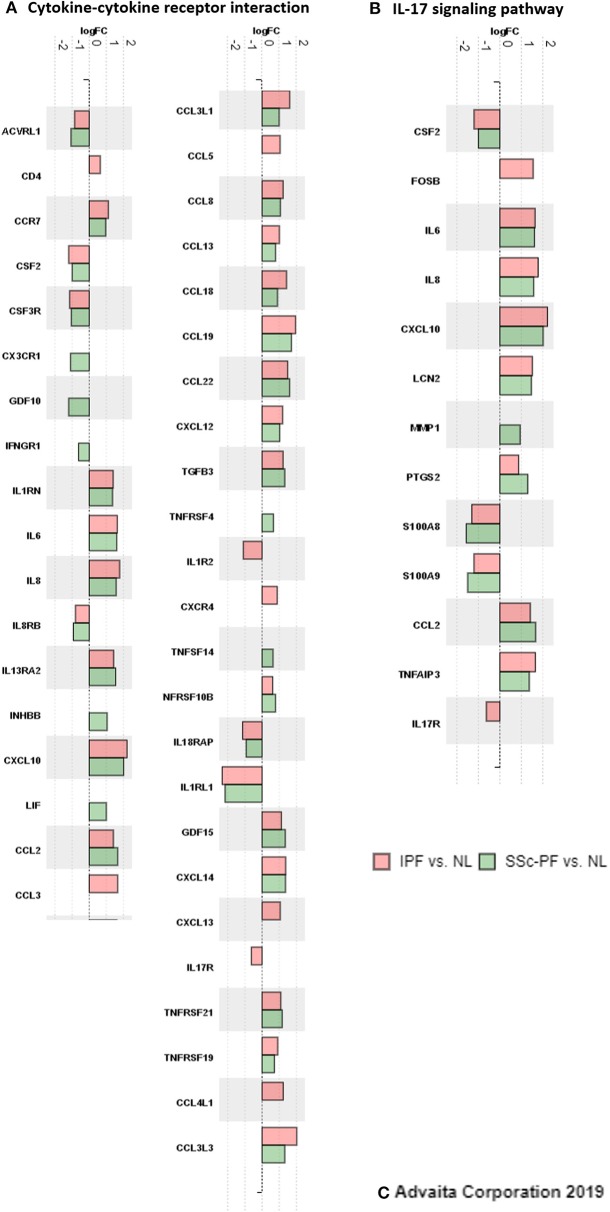
Inflammatory response signatures in IPF and SSc-PF. DE genes (*q* < 0.1, log2FC > |0.6|, linear FC of 1.5) that are hit genes in the “cytokine-cytokine receptor interaction” and “IL-17 signaling” pathways are shown here, color-coded by DE analysis. Peach: IPF vs. NL, and green: SSc-PF vs. NL.

Next, we aimed to tease out similarities and differences in the inflammatory response signature of both diseases by specifically looking at two biological pathways present in the intersect: “Cytokine-cytokine receptor interaction” and “IL-17 signaling pathway” (Figure 8 and Supplementary Figures 8–11). At first glance, the inflammatory response in both diseases looked very similar and nearly all of the hit genes in these pathways were differentially expressed in both diseases with the same direction of regulation. However, subtle differences were captured, especially in the pathway impact analysis that revealed perturbations and coherent cascades. Specifically, in the SSc-PF “cytokine-cytokine receptor interaction” pathway under TNF Family (Supplementary Figure 8), *TNFSF14*-associated perturbation (labeled LIGHT) that leads to upregulation of *LTBR, HVEM*, and *DCR3* was not observed in IPF (Supplementary Figure 10). Additionally, upregulation of LIF that cascades to upregulation of *LIFR* and *IL6ST* was exclusive to SSc-PF. In the “IL-17” signaling pathway, all DE chemokines, cytokines and anti-microbial genes were the same in both diseases (Supplementary Figures 9, 11), but upstream of this cascade *IL-17RA* and *FOSB* (labeled *API*) were only differentially expressed in IPF (Supplementary Figure 11). Together these findings revealed overall similar inflammatory responses in IPF and SSc-PF with subtle differences in TNF and IL6/IL12/IL17 signaling pathways.

## Discussion

SSc-PF and IPF are distinct diseases but they do share several pathobiologic characteristics (5). The goal of this study was to (1) characterize the similarities existing in the development of pulmonary fibrosis in scleroderma and IPF to find potential therapeutic targets that could be beneficial in both diseases, and (2) to tease out the exclusive signature that each disease has, using a systems level and protein-protein interaction network approach. In both diseases, inflammation and immune responses were highly enriched, revealing that even at an advanced stage of pulmonary fibrosis, these “first-response” feedback mechanisms are still active and on-going.

### The Intersection Signature Highlights Potential Therapeutic Targets Beneficial for Both SSc-PF and IPF

The systems level analysis we performed on the DE genes in the intersection between IPF and SSc-PF was dominated by “immune response,” “collagen metabolism,” and “ECM organization” super clusters (Figure 3) and most of the query genes entered in the network were hit genes in “immune system processes” term, including hub proteins IL6, COL1A1, COL1A2, VCAM1, CXCL10, CXCL12, CCL2, CCL19, and FGG. Our data also showed that the IGF family members *IGFBP2* and *IGFL2* are significantly upregulated in both SSc-PF and IPF lung tissues, and that the coagulation cascade was a prominent protein-protein interaction sub-network in the intersection signature.

#### Interleukin 6 (IL6) and Its Role in Autoimmunity, Inflammation, and Fibrogenesis

The IL17 signaling pathway is critical for the activation of inflammatory genes and production of chemokines and cytokines such as IL6 (Supplementary Figures 9, 11) (34). IL6 is a multi-faceted pro-inflammatory cytokine synthesized by fibroblasts, peripheral blood mononuclear cells (PBMCs), B cells, macrophages, dendritic cells, monocytes and mast cells, that plays a role in chronic inflammation, autoimmunity, endothelial cell dysfunction, vascularization/angiogenesis and fibrogenesis (35–41). We characterized *IL6* as a hub protein upregulated in both IPF and SSc-PF tissues (Supplementary Table 3) that has a strong influence on the immune response. *IL6* is overexpressed in dermal fibroblasts, PBMCs, mononuclear and endothelial cells of SSc patients (37, 38, 42). Interestingly, few effector cells express the functional membrane IL6 receptor (IL6R); lymphocytes, hepatocytes, monocytes, B cells, neutrophils and a subset of T-cells (35). Other cell types that do not express IL6R (i.e., fibroblasts and endothelial cells) can still respond to IL6 due to trans-signaling of soluble IL6R (sIL6R) and gp130 receptor (39).

In both SSc-PF and IPF, the IL17 signaling pathway is enriched (Supplementary Figures 9, 11) and the expression of the ubiquitin-editing enzyme A20 (TNFAIP3), a major regulator of NFκB activation, is upregulated (43, 44). A20 reduces NFκB activation by inhibiting TNF receptor signaling and removing K63-linked ubiquitin chains conjugated to TRAF6 (45, 46). The observed upregulation of A20 suggests downregulation of the IL17 signaling pathway (47). However, several chemokines and cytokines downstream of IL17 signaling were upregulated (IL6, CXCL8, CXCL10, CCL2, and COX2), indicating that other regulatory mechanisms are likely at play that affect the expression of downstream genes in this pathway. Inhibition of TNFα signaling has shown promising results in many patients with inflammatory disorders (48), but has also generated adverse side effects (49), highlighting that TNFα signaling is a double-edged sword that can be both pro- and anti-inflammatory.

Serum IL6 is a predictive marker of early functional decline and mortality in SSc-PF (50). Here we found that *IL6* levels are still significantly increased in advanced SSc-PF and IPF, suggesting that IL6 lingers to stimulate more collagen synthesis and chronic inflammation, consistent with Koch et al. (51). Activated lung macrophages have also been shown to enhance IL6 trans-signaling via ADAM-17 leading to fibroblast proliferation and ECM deposition, and inhibition of sIL6Rα can attenuate pulmonary inflammation and fibrosis (52). In recent clinical trials, blocking IL6 trans-signaling using the monoclonal antibody tocilizumab showed promising trends in patients with SSc (53), suggesting that IL6 might be a viable therapeutic target in SSc. Our current findings also suggest that targeting IL6 trans-signaling may be a suitable therapeutic approach for the treatment of IPF.

#### Targeting IGFBP2 and IGFL2

We show here that the IGF family members *IGFBP2* and *IGFL2* are significantly upregulated in both SSc-PF and IPF lungs tissues. IGFBP2 is secreted into the bloodstream where it binds IGF1 and IGF2 with high affinity, can interact with several different ligands, and can be localized intracellularly (54). Guiot et al. showed that IGFBP2 levels are increased in serum and sputum samples of IPF patients, and characterized IGFBP2 as a biomarker of IPF severity (55, 56). In patients with diffuse cutaneous SSc (dcSSc), IGFBP2 levels are also increased in both serum and skin biopsies (57). Additionally, IGFBP2 is abundant in collagen1-stimulated peripheral blood mononuclear cells (PBMCs) from juvenile and adult dcSSc patients (58).

Findings with IGFBP2 are consistent with our previous studies showing increased expression of *IGFBP3* and *IGFBP5* in IPF and SSc-PF (25, 26) and reinforced the crucial role that IGFBPs and the IGF pathway play in the development and perpetuation of pulmonary fibrosis (22–24). Note however that IGFBPs can also signal via membrane bound proteins such as integrins in a IGF-independent pathway (59). We previously showed that IGFBP5 induces fibrosis by promoting fibroblast activation into myofibroblasts and recruitment of pro-inflammatory cells (60, 61) via p44/p42 mitogen-activated protein kinase (MAPK) pathway and induction of EGR1 and DOK5 (62, 63). Additionally, IGFBP5 induces the expression of collagen 1, fibronectin, CTGF and lysyl oxidase, as well as its own expression by positive feedback mechanism (26).

IGFBP3 induces tenascin C (TNC), a biomarker of SSc-PF (3). Importantly, both IGFBP3 and IGFBP5 contribute to extracellular (ECM) deposition in IPF (25) and promote fibrosis in human skin maintained in organ culture (64), demonstrating direct relevance to the human disease. Targeting IGFBPs that are elevated in SSc-PF and IPF is an appealing therapeutic strategy yet no clinical trials employing this approach have been reported.

*IGFL2* expression is also increased in SSc-PF and IPF, however its biological function is not well-characterized. IGFL2 does not have a transmembrane domain, it is found in secreted form in the ECM, and it may play a critical role in cellular energy metabolism as well as in growth and development (65). In human skin fibroblasts undergoing mitochondrial depletion, *IGFL2* levels are substantially decreased (dataMED, ID: E-GEOD-24945), suggesting a connection between mitochondrial physiology and IGFL2 secretion. Its specific role in SSc-PF and IPF remains to be elucidated.

#### Collagen-Derived Matrikines

Collagen-derived matrikines are peptides generated by partial proteolysis of certain collagens that can regulate cell activity (30). Out of the 9 DE collagen genes identified in the intersection of SSc-PF and IPF, 4 have been identified as sources of matrikines: *COL1A1, COL1A2, COL15A1*, and *COL17A1* (30, 66, 67).

Two matrikines are derived from COL1A1: DGGRYY peptide that can inhibit human neutrophil activation by collagen (68), and COL1 matricryptin p1158/1159 that has recently shown promising results in generating new ECM and stimulating angiogenesis post myocardial infarction (69).

The tripeptide GHK is produced from cleavage of COL1A2 and forms the complex GHK-Cu due to its high affinity to copper ions (30). This complex regulates a plethora of biological processes relevant for ECM remodeling: (1) chemoattraction of repair cells, (2) anti-inflammation, (3) synthesis of collagen, elastin, MMPs, anti-proteases, VEGF, FGF2, NGF, and EPO, (4) stimulation of angiogenesis, and (5) proliferation of fibroblasts and keratinocytes (70). Recently, the GHK-Cu complex was shown to inhibit bleomycin-induced PF in mice by suppressing TGFβ1/Smad-mediated epithelial to mesenchymal transition (71).

Restin is a COL15-derived matrikine that can inhibit endothelial cell proliferation and has anti-angiogenic properties similar to endostatin, with the latter being a cleavage product of COL18 (72, 73).

COL17A1 sheds a soluble triple-helical ectodomain (aka BP180) under the regulation of ADAM9 and ADAM10 (67, 74). Less is known about the function of BP180. BP180 acts as a core anchor protein due to its multiple binding sites (i.e., extracellular domains of integrin α6, cytoplasmic domains of integrin β4, laminin-5 and plectin) connecting intra- and extra-cellular hemidesmosomal proteins and playing a role in the development of bullous pemphigoid (75). Cleavage of BP180 enhances neutrophil chemotaxis and initiates the release of inflammatory factors (75, 76).

#### Fibrinogen Hub Proteins

Fibrinogen proteins FGA, FGB, and FGG are essential for a variety of processes, including blood clot formation, wound healing, inflammation and blood vessel growth (77). When fibrinogen is cleaved by thrombin into insoluble fibrin polymer, the formation of a fibrin clot occurs, a process that is essential for hemostasis and wound healing. As part of the fibrinolytic system, fibrin binds and cleaves plasminogen into plasmin, leading to fibrin digestion and removal of fibrin clots.

In this study, we found that FGG was a hub protein in the protein-protein network interacting with IL6, FGA, FGB, CDH2, CPB2, SPP1, TGFβ3, and SERPIND1 that was significantly downregulated in both IPF and SSc-PF lung tissues (Figure 3), in agreement with recent work by Vukmirovic et al. (78). During PF, impaired coagulation cascade plays a role in orchestrating inflammatory and tissue repair responses as well as fibrogenesis via activation of proteinase-activated receptors (79). Additionally, the fibrinolytic pathway has been shown to have anti-fibrotic actions in PF thanks to COX2 induction by plasminogen leading to prostaglandin E2 synthesis and repression of collagen expression (80).

#### TLR8: Autoimmunity, Fibrosis, and Angiogenesis

Because the respiratory airways are constantly exposed to environmental pathogens and other elements, TLRs play a crucial role in innate immunity to endogenous and exogenous ligands (81). TLR2 and TLR4 have been shown to trigger immune and fibrotic responses in PF (82). TLR4 activation induces the release of profibrotic and proangiogenic chemokines from anti-fibroblast antibodies stimulated fibroblasts (83). Furthermore, activation of TLR4 signaling in skin and lung fibroblasts increased TGFβ1 sensitivity and ECM production, both processes contributing to persistent fibrogenesis in SSc (84).

In this study, we determined that TLR8 was a hub protein in the SSc-PF network (Figure 4) that was downregulated at the transcript level in both SSc-PF and IPF, albeit not significantly. Another study examining TLR expression levels in bronchoalveolar lavage fluid of patients with IPF and fibrotic interstitial pneumonias associated with collagen tissue disorders reported no significant difference in TLR8 levels in these 2 patient cohorts as compared to a control group (85). The role of TLR8 during the development of SSc-PF remains elusive, but TLR8 activation has been shown to promote inflammatory responses and fibrogenesis in skin fibrosis and lung injury (86, 87). Together this suggests that TLR8 expression may be upregulated in early and intermediate stages of PF when inflammation is more prominent, and its activation contributes to enhanced inflammation and fibrogenesis, whereas TLR8 expression returns to low levels in late stages of PF, when inflammation is less evident. In the skin of SSc patients, the infiltration and activation of plasmacytoid dendritic cells (pDCs) via PI3Kδ pathway leads to aberrant expression of TLR8 (otherwise not expressed in pDCs) that induces CXCL4, IFN-α, IL6, and TNF secretion contributing to skin fibrosis and autoimmunity (87). Inhibitors of PI3Kδ have been approved for treatment of cancer, inflammatory and autoimmune diseases (88), and our data provide rationale to further explore the targeting of the PI3Kδ-TLR8 axis in SSc-PF and IPF, via repurposing drugs used for cancer treatment.

TLR8 is a gene hit in the “Regulation of localization” biological process and has protein-protein interaction with other hub proteins including PTGS2, MPO, and ARG1 (Figure 4). In SSc-PF lungs, *TLR8, MPO* and *ARG1* are downregulated while *PTGS2* is upregulated (Supplementary Table 4). Expression profiling for these 4 hub proteins may indeed capture advanced stages of fibrosis in SSc-PF lungs, emphasizing that the damage caused by inflammation and fibrosis is so far advanced that lung transplantation is necessary and justified.

### The Unique Transcriptomic Signature in SSc-PF Relates to Vasculature Development and Perturbation of Bioenergetics

Even though SSc-PF and IPF have common characteristics, SSc-PF has a unique transcriptomic signature and features (5). Here we found that “regulation of vasculature development” was a Biological Process super cluster in the GO analysis of DE genes unique to SSc-PF, and PTGS2, ARG1 and EDN1 were related hub proteins under the subcategories “tube development” and “blood circulation.” This is consistent with vascular complications and angiogenesis impairment being trademarks in SSc patients (89), and with structural disintegration of vasculature and loss of endothelial cell numbers observed in late stage of SSc-PF (2).

#### ARG1

ARG1 is a key enzyme in the urea cycle that converts L-arginine to L-ornithine, which is further metabolized into proline and polyamines, drivers of collagen synthesis (90). ARG1 is also a marker of activated macrophages (M2), producers of angiogenic factors (91, 92). Macrophages have been shown to regulate the rate of conversion to proline and IL4Rα-induced stimulation of macrophages increases ARG1, proline output and fibrosis sequentially (93). Targeting ARG1 metabolism is an emerging therapeutic strategy in the treatment of inflammation-induced suppression of T lymphocyte proliferation (immunosuppression) (94).

#### PTGS2 (Aka COX2)

Cyclooxygenase (COX) enzymes contribute to the release of lipid mediators in arachidonic acid metabolism, and PTGS2/COX2 plays a key role not only in prostaglandin signaling, but also in the fibrinolytic pathway and fibrogenesis, as well as in inflammatory cytokine-induced angiogenesis (80, 95). *PTGS2/COX2* was exclusively upregulated in SSc-PF lung tissues (Supplementary Table 4) and is a hub protein that interacts with TLR8, IRF1, ARG1, CAMP, LIF, MMP10, RCAN1, GGT1, EDN1, and MPO. Prostaglandin signaling has been shown to promote PF independently of TGFβ and loss of the prostaglandin F receptor reduced fibrosis in the bleomycin-induced PF model without affecting alveolar inflammation (96). Additionally, *PTGS2/COX2* selective inhibitors markedly reduced IL1β-induced angiogenesis *in vivo* (95). Together these data suggest that targeting *PTGS2/COX2* with a therapeutic drug could modulate both fibrosis and angiogenesis in SSc patients.

#### Overall Perturbation of FA Metabolism and Glycolysis in SSc-PF

Altered cellular bioenergetics as a driving force behind fibrogenesis is an emerging field (97–100). Here we found that FA metabolism (FA degradation and biosynthesis of unsaturated FA) and glycolysis pathways were significantly perturbed in SSc-PF lungs.

Metabolic reprogramming is a hallmark of cancer that is also observed during immune response and inflammation during which glycolysis becomes the alternative pathway to oxidative phosphorylation for ATP production (101, 102). Interestingly, inhibition of glycolysis has been shown to attenuate lung fibrosis (103), suggesting that metabolic reprogramming also plays a crucial role in the development of PF. Additionally, Selvarajah et al. (99) concluded that glycolysis (but not mitochondrial respiration) was necessary for TGFβ1-induced collagen deposition in primary human lung fibroblasts. This may also be true for other cell types that are present in whole lung tissue.

Fluctuation between glycolysis and fatty acid oxidation (FAO) has been shown to govern macrophages and dendritic cells as well as fibroblasts during inflammation and ECM remodeling, respectively (100, 102). In fact, the FA transporter CD36 has been identified as crucial mediator of COL1 internalization and degradation that can be targeted to reduce murine skin fibrosis (100). Our data show that biosynthesis of unsaturated FA and FA degradation, the steps that ultimately generate acetyl-CoA required for the citric acid cycle to produce energy within mitochondria, are significantly deregulated in SSc-PF. Interestingly, this signature has also been uncovered in renal epithelial cells from patients with kidney fibrosis (104). Additionally, in TGFβ1-induced fibrotic models, suppression of FAO and enhancement of glycolysis are observed (99, 104), highlighting that a shift in bioenergetics, similar to the Warburg effect characteristic of cancer cells, is ongoing during fibrogenesis.

In all, metabolic reprogramming appears to hijack energy production of several cell types that are the drivers of inflammation, fibrogenesis and ECM remodeling, disturbing the homeostatic balance between glycolysis and FAO, ultimately shifting the cellular fuel source and activating pathological processes. Once key players regulating this reprogramming are identified, the development of anti-fibrotic therapies targeting metabolic molecules will be possible. Such potential therapeutic targets have already been identified and tested in experimental PF models (100, 103).

## Conclusion

By conducting differential expression analyses on lung tissues from IPF and SSc-PF, we were able to compare the gene signature of these two pulmonary fibrotic diseases that share many pathobiologic features and identify shared features as well as exclusive characteristics for each disease. Our data suggest that therapeutic strategies targeting IL6 trans-signaling, *IGFBP2, IGFL2*, and the coagulation cascade may be efficacious in both SSc-PF and IPF. In SSc-PF, additional potential targets include mediators of fatty acid metabolism and glycolysis as well as *TLR8*.

## Data Availability Statement

The datasets generated for this study can be found in the NCBI GEO GSE48149.

## Ethics Statement

The studies involving human participants were reviewed and approved by the University of Pittsburgh Institutional Review Board. The patients/participants provided their written informed consent to participate in this study.

## Author Contributions

Study design and manuscript editing: LR and CF-B. Differential expression analysis (microarray)—gene level analysis: WS, LR, and GH. Systems level analysis and protein-protein interaction network: LR. Validation qRT-PCR: NT. Writing of the manuscript and generation of figures with statistics and heatmaps: LR, NT, and CF-B. Reviewing the draft and comments: WS, NT, and GH.

### Conflict of Interest

The authors declare that the research was conducted in the absence of any commercial or financial relationships that could be construed as a potential conflict of interest. The handling editor declared a past co-authorship with one of the authors, CF-B.
