# Supplementary material for: Prominence of IL6, IGF, TLR, and Bioenergetics Pathway Perturbation in Lung Tissues of Scleroderma Patients With Pulmonary Fibrosis
Source: Front Immunol. 2020 Mar 10;11:383. doi: 10.3389/fimmu.2020.00383 (PMC7075854; doi:10.3389/fimmu.2020.00383)
Supplement: Supplementary file 1 [file Table_1.DOCX]

***Supplementary Table 1***

Prominence of IL6, IGF, TLR and bioenergetics pathway perturbation in lung tissues of scleroderma patients with pulmonary fibrosis

**Ludivine Renaud****^1^, Willian A. da Silveira^2^, Naoko Takamura^1^, Gary Hardiman^2^, Carol Feghali-Bostwick^1^***

^1^ Department of Medicine, Medical University of South Carolina, Charleston, SC, USA.

^2^ School of Biological Sciences and Institute for Global Food Security, Queens University Belfast, Belfast BT9 5AG, UK.

*** Correspondence:**Dr. Carol Feghali-Bostwick
feghalib@musc.edu

**Supplementary Table 1**: **List of DE genes in SSc-PF tissue**. The criteria for significance are q<0.1, logFC>1: upregulated (in red), logFC<-1: downregulated (in blue). Data is sorted on q-value from smallest to highest.

| **ID_REF** | **NCBI_Reference** | **HUGO_Symbol** | **Entrez_ID** | **AveExpr** | **t** | **B** | **logFC** | **q-value** |
| --- | --- | --- | --- | --- | --- | --- | --- | --- |
| ILMN_4766 | NM_002391.3 | MDK | 4192 | 9.73 | 11.26 | 19.35 | 1.65 | 8.58E-09 |
| ILMN_5174 | NM_173485.2 | ZNF218 | 128553 | 10.56 | 10.17 | 16.85 | 1.39 | 6.39E-08 |
| ILMN_27702 | NM_053279.1 | C8ORF13 | 83648 | 8.88 | -10.01 | 16.48 | -1.44 | 6.39E-08 |
| ILMN_176067 | NM_001042500.1 | LOC653600 | 728358 | 8.23 | -9.32 | 14.79 | -2.82 | 2.92E-07 |
| ILMN_137905 | NM_002084.2 | GPX3 | 2878 | 14.41 | -9.10 | 14.23 | -1.40 | 3.92E-07 |
| ILMN_22426 | NM_013279.1 | C11ORF9 | 745 | 8.11 | -9.06 | 14.13 | -1.68 | 3.92E-07 |
| ILMN_12837 | NM_006579.1 | EBP | 10682 | 10.32 | -9.00 | 13.96 | -1.10 | 4.00E-07 |
| ILMN_22736 | NM_006288.2 | THY1 | 7070 | 11.30 | 8.79 | 13.44 | 2.78 | 5.41E-07 |
| ILMN_9651 | NM_014452.3 | TNFRSF21 | 27242 | 12.73 | 8.65 | 13.08 | 1.17 | 7.17E-07 |
| ILMN_12740 | NM_001793.3 | CDH3 | 1001 | 10.18 | 8.46 | 12.57 | 2.11 | 8.81E-07 |
| ILMN_20831 | NM_001450.3 | FHL2 | 2274 | 10.42 | 8.46 | 12.57 | 1.85 | 8.81E-07 |
| ILMN_21050 | NM_001007023.1 | DIO2 | 1734 | 8.67 | 8.37 | 12.33 | 1.60 | 9.54E-07 |
| ILMN_11220 | NM_005217.2 | DEFA3 | 1668 | 8.63 | -8.36 | 12.31 | -3.09 | 9.54E-07 |
| ILMN_6950 | NM_003020.1 | SCG5 | 6447 | 8.93 | 8.11 | 11.65 | 1.65 | 1.71E-06 |
| ILMN_7203 | NM_002935.2 | RNASE3 | 6037 | 7.21 | -8.12 | 11.68 | -1.03 | 1.71E-06 |
| ILMN_828 | NM_080878.2 | ITLN2 | 142683 | 8.92 | -8.03 | 11.42 | -3.47 | 2.07E-06 |
| ILMN_24078 | NM_003013.2 | SFRP2 | 6423 | 11.42 | 7.89 | 11.04 | 2.45 | 2.46E-06 |
| ILMN_27777 | NM_014573.1 | TMEM97 | 27346 | 10.85 | -7.89 | 11.06 | -1.34 | 2.46E-06 |
| ILMN_25111 | NM_000693.1 | ALDH1A3 | 220 | 11.35 | 7.83 | 10.91 | 2.31 | 2.65E-06 |
| ILMN_20370 | NM_005261.2 | GEM | 2669 | 9.49 | 7.83 | 10.90 | 1.47 | 2.65E-06 |
| ILMN_29692 | NM_004084.2 | DEFA1 | 1667 | 9.22 | -7.80 | 10.81 | -3.50 | 2.81E-06 |
| ILMN_12246 | NM_001001438.1 | LSS | 4047 | 9.97 | -7.76 | 10.69 | -1.04 | 3.07E-06 |
| ILMN_163190 | NM_015993.1 | TM4SF11 | 51090 | 10.46 | -7.69 | 10.50 | -1.98 | 3.50E-06 |
| ILMN_4697 | NM_014696.2 | KIAA0514 | 9721 | 9.41 | -7.64 | 10.38 | -1.50 | 3.85E-06 |
| ILMN_11594 | NM_012261.2 | C20ORF103 | 24141 | 8.91 | 7.62 | 10.32 | 1.88 | 3.99E-06 |
| ILMN_975 | NM_018945.3 | PDE7B | 27115 | 9.55 | 7.45 | 9.85 | 1.02 | 6.00E-06 |
| ILMN_2390 | NM_023930.2 | KCTD14 | 65987 | 9.78 | -7.40 | 9.73 | -1.25 | 6.53E-06 |
| ILMN_2640 | NM_022351.2 | EFCBP1 | 64168 | 7.43 | -7.35 | 9.58 | -1.06 | 7.22E-06 |
| ILMN_10023 | NM_005525.2 | HSD11B1 | 3290 | 9.58 | -7.35 | 9.57 | -1.15 | 7.22E-06 |
| ILMN_139058 | NM_005161.2 | AGTRL1 | 187 | 9.74 | 7.31 | 9.46 | 2.33 | 7.92E-06 |
| ILMN_12582 | NM_134421.1 | HPCAL1 | 3241 | 11.57 | -7.25 | 9.30 | -1.18 | 8.94E-06 |
| ILMN_5566 | NM_000422.1 | KRT17 | 3872 | 11.13 | 7.14 | 9.01 | 3.48 | 1.13E-05 |
| ILMN_138379 | NM_004055.3 | CAPN5 | 726 | 9.92 | 7.14 | 8.99 | 1.03 | 1.13E-05 |
| ILMN_18400 | NM_001925.1 | DEFA4 | 1669 | 7.78 | -7.09 | 8.85 | -1.17 | 1.24E-05 |
| ILMN_20021 | NM_032638.3 | GATA2 | 2624 | 9.57 | -7.09 | 8.84 | -1.45 | 1.24E-05 |
| ILMN_21257 | NM_021158.3 | TRIB3 | 57761 | 9.67 | -7.07 | 8.79 | -1.08 | 1.26E-05 |
| ILMN_16252 | NM_001878.2 | CRABP2 | 1382 | 9.02 | 7.04 | 8.71 | 1.50 | 1.35E-05 |
| ILMN_4927 | NM_024508.3 | ZBED2 | 79413 | 8.48 | -7.02 | 8.65 | -2.06 | 1.41E-05 |
| ILMN_26228 | NM_003358.1 | UGCG | 7357 | 10.93 | 6.93 | 8.42 | 1.28 | 1.56E-05 |
| ILMN_24828 | NM_001852.3 | COL9A2 | 1298 | 9.29 | 6.85 | 8.19 | 1.12 | 1.91E-05 |
| ILMN_20095 | NM_206930.1 | SYTL2 | 54843 | 9.91 | 6.85 | 8.18 | 1.03 | 1.91E-05 |
| ILMN_12928 | NM_004419.3 | DUSP5 | 1847 | 11.30 | 6.82 | 8.10 | 1.63 | 2.03E-05 |
| ILMN_18738 | NM_013402.3 | FADS1 | 3992 | 10.22 | -6.80 | 8.03 | -1.07 | 2.14E-05 |
| ILMN_2732 | NM_000597.2 | IGFBP2 | 3485 | 11.58 | 6.79 | 8.01 | 1.50 | 2.16E-05 |
| ILMN_11545 | NM_001136.3 | AGER | 177 | 10.24 | -6.78 | 7.98 | -2.77 | 2.18E-05 |
| ILMN_12517 | NM_022746.2 | MOSC1 | 64757 | 9.95 | -6.77 | 7.95 | -1.18 | 2.19E-05 |
| ILMN_18980 | NM_002130.4 | HMGCS1 | 3157 | 11.69 | -6.77 | 7.95 | -1.27 | 2.19E-05 |
| ILMN_11566 | NM_000240.2 | MAOA | 4128 | 13.46 | -6.67 | 7.69 | -1.07 | 2.78E-05 |
| ILMN_16923 | NM_000396.2 | CTSK | 1513 | 11.26 | 6.65 | 7.62 | 1.40 | 2.93E-05 |
| ILMN_5749 | NM_006163.1 | NFE2 | 4778 | 9.16 | -6.63 | 7.57 | -1.79 | 2.96E-05 |
| ILMN_16950 | NM_024728.1 | C7ORF10 | 79783 | 8.67 | 6.61 | 7.50 | 1.32 | 3.11E-05 |
| ILMN_24830 | NM_000094.2 | COL7A1 | 1294 | 10.93 | 6.59 | 7.45 | 2.14 | 3.20E-05 |
| ILMN_19972 | NM_020384.2 | CLDN2 | 9075 | 8.27 | 6.58 | 7.42 | 1.96 | 3.28E-05 |
| ILMN_12396 | NM_173642.1 | FAM80A | 284716 | 8.15 | -6.52 | 7.25 | -1.02 | 3.71E-05 |
| ILMN_11215 | NM_002686.2 | PNMT | 5409 | 7.77 | -6.51 | 7.21 | -1.20 | 3.77E-05 |
| ILMN_15049 | NM_000095.2 | COMP | 1311 | 10.88 | 6.47 | 7.10 | 2.59 | 4.20E-05 |
| ILMN_9853 | NR_002766.1 | MEG3 | 55384 | 10.80 | 6.43 | 6.99 | 1.32 | 4.66E-05 |
| ILMN_15732 | NM_001855.2 | COL15A1 | 1306 | 11.24 | 6.39 | 6.87 | 1.96 | 5.14E-05 |
| ILMN_16128 | NM_004104.4 | FASN | 2194 | 12.78 | -6.38 | 6.84 | -1.40 | 5.15E-05 |
| ILMN_20087 | NM_000494.2 | COL17A1 | 1308 | 8.94 | 6.36 | 6.81 | 2.06 | 5.21E-05 |
| ILMN_24855 | NM_021101.3 | CLDN1 | 9076 | 11.38 | 6.35 | 6.77 | 1.96 | 5.21E-05 |
| ILMN_4355 | NM_024579.1 | C1ORF54 | 79630 | 11.78 | 6.37 | 6.82 | 1.07 | 5.21E-05 |
| ILMN_7361 | NM_007288.1 | MME | 4311 | 8.37 | -6.36 | 6.79 | -1.12 | 5.21E-05 |
| ILMN_18011 | NM_152547.2 | BTNL9 | 153579 | 8.67 | -6.36 | 6.80 | -2.03 | 5.21E-05 |
| ILMN_17467 | NM_180991.4 | SLCO4C1 | 353189 | 8.53 | -6.31 | 6.66 | -1.31 | 5.73E-05 |
| ILMN_28982 | NM_015170.1 | SULF1 | 23213 | 10.72 | 6.30 | 6.61 | 1.55 | 5.97E-05 |
| ILMN_138827 | NM_145810.1 | CDCA7 | 83879 | 8.54 | 6.29 | 6.59 | 1.22 | 6.00E-05 |
| ILMN_930 | NM_001037500.1 | DEFB124 | 245937 | 8.16 | 6.28 | 6.58 | 1.06 | 6.00E-05 |
| ILMN_29922 | NM_015419.1 | MXRA5 | 25878 | 11.86 | 6.28 | 6.55 | 1.58 | 6.09E-05 |
| ILMN_13811 | NM_003239.1 | TGFB3 | 7043 | 10.77 | 6.25 | 6.49 | 1.32 | 6.38E-05 |
| ILMN_21395 | NM_002990.3 | CCL22 | 6367 | 8.85 | 6.23 | 6.42 | 1.61 | 6.57E-05 |
| ILMN_17477 | NM_032199.1 | ARID5B | 84159 | 12.33 | 6.20 | 6.33 | 1.03 | 7.08E-05 |
| ILMN_27165 | NM_015715.2 | PLA2G3 | 50487 | 9.03 | -6.18 | 6.29 | -1.36 | 7.22E-05 |
| ILMN_11485 | NM_177403.3 | RAB7B | 338382 | 10.14 | 6.14 | 6.15 | 1.29 | 7.94E-05 |
| ILMN_36330 | XM_941554.1 | KIAA1912 | 114800 | 8.90 | -6.13 | 6.14 | -1.45 | 7.98E-05 |
| ILMN_5522 | NM_001031692.1 | LRRC17 | 10234 | 8.66 | 6.10 | 6.05 | 1.16 | 8.60E-05 |
| ILMN_19248 | NM_000954.5 | PTGDS | 5730 | 13.03 | 6.08 | 6.00 | 1.28 | 9.01E-05 |
| ILMN_9188 | NM_002423.3 | MMP7 | 4316 | 12.41 | 6.07 | 5.95 | 3.38 | 9.26E-05 |
| ILMN_5003 | NM_032641.1 | SPSB2 | 84727 | 9.65 | -5.97 | 5.67 | -1.07 | 1.19E-04 |
| ILMN_20214 | NM_000640.2 | IL13RA2 | 3598 | 8.47 | 5.96 | 5.65 | 1.55 | 1.20E-04 |
| ILMN_9394 | NM_000582.2 | SPP1 | 6696 | 11.30 | 5.92 | 5.53 | 3.67 | 1.35E-04 |
| ILMN_26240 | NM_001257.3 | CDH13 | 1012 | 8.17 | -5.90 | 5.47 | -1.02 | 1.38E-04 |
| ILMN_19494 | NM_000045.2 | ARG1 | 383 | 7.54 | -5.90 | 5.48 | -1.03 | 1.38E-04 |
| ILMN_11548 | NM_000088.2 | COL1A1 | 1277 | 12.84 | 5.89 | 5.43 | 2.09 | 1.42E-04 |
| ILMN_26242 | NM_019609.3 | CPXM | 56265 | 9.05 | 5.88 | 5.42 | 1.70 | 1.42E-04 |
| ILMN_16792 | NM_021935.2 | PROK2 | 60675 | 8.94 | -5.88 | 5.42 | -2.15 | 1.42E-04 |
| ILMN_24337 | NM_078488.1 | VNN2 | 8875 | 9.02 | -5.85 | 5.34 | -1.27 | 1.47E-04 |
| ILMN_138322 | NM_007350.2 | PHLDA1 | 22822 | 12.12 | 5.78 | 5.12 | 1.41 | 1.72E-04 |
| ILMN_21728 | NM_033131.2 | WNT3A | 89780 | 8.42 | -5.76 | 5.08 | -1.13 | 1.78E-04 |
| ILMN_2040 | NM_012109.1 | C19ORF4 | 25789 | 8.56 | 5.74 | 5.02 | 1.19 | 1.88E-04 |
| ILMN_1899 | NM_139212.2 | HOP | 84525 | 12.17 | -5.72 | 4.96 | -1.20 | 1.98E-04 |
| ILMN_13399 | NM_001032278.1 | MMP28 | 79148 | 9.73 | -5.71 | 4.93 | -1.06 | 2.00E-04 |
| ILMN_16399 | NM_006086.2 | TUBB3 | 10381 | 9.32 | 5.67 | 4.82 | 1.41 | 2.19E-04 |
| ILMN_2442 | NM_004772.1 | C5ORF13 | 9315 | 10.91 | 5.62 | 4.67 | 1.30 | 2.52E-04 |
| ILMN_174163 | NM_022153.1 | C10ORF54 | 64115 | 10.45 | -5.56 | 4.49 | -1.01 | 2.89E-04 |
| ILMN_25235 | NM_016206.2 | VGLL3 | 389136 | 9.37 | -5.56 | 4.49 | -1.16 | 2.89E-04 |
| ILMN_2977 | NM_014505.4 | KCNMB4 | 27345 | 8.32 | -5.55 | 4.47 | -1.12 | 2.93E-04 |
| ILMN_3827 | NM_004093.2 | EFNB2 | 1948 | 11.04 | -5.53 | 4.41 | -1.04 | 3.07E-04 |
| ILMN_7308 | NM_005265.2 | GGT1 | 2678 | 8.52 | -5.52 | 4.38 | -1.07 | 3.16E-04 |
| ILMN_1036 | NM_004000.2 | CHI3L2 | 1117 | 9.40 | -5.51 | 4.37 | -1.48 | 3.17E-04 |
| ILMN_26397 | NM_003725.2 | HSD17B6 | 8630 | 10.52 | -5.49 | 4.28 | -1.90 | 3.35E-04 |
| ILMN_7429 | NM_001701.2 | BAAT | 570 | 7.89 | 5.46 | 4.20 | 1.14 | 3.51E-04 |
| ILMN_170763 | NM_001042459.1 | FILIP1L | 11259 | 11.46 | 5.46 | 4.21 | 1.03 | 3.51E-04 |
| ILMN_9299 | NM_014392.2 | D4S234E | 27065 | 8.98 | 5.43 | 4.11 | 1.48 | 3.82E-04 |
| ILMN_27995 | NM_018058.4 | CRTAC1 | 55118 | 10.39 | -5.40 | 4.05 | -1.87 | 4.02E-04 |
| ILMN_27413 | NM_144707.1 | PROM2 | 150696 | 10.07 | 5.39 | 4.01 | 2.00 | 4.09E-04 |
| ILMN_7268 | NM_153345.1 | FLJ90586 | 135932 | 8.04 | -5.38 | 3.99 | -1.02 | 4.11E-04 |
| ILMN_14815 | NM_004170.4 | SLC1A1 | 6505 | 8.63 | -5.36 | 3.92 | -1.14 | 4.32E-04 |
| ILMN_5029 | NM_000071.1 | CBS | 875 | 9.54 | -5.35 | 3.90 | -1.20 | 4.35E-04 |
| ILMN_27781 | NM_014476.1 | PDLIM3 | 27295 | 11.87 | 5.35 | 3.89 | 1.10 | 4.36E-04 |
| ILMN_6606 | NM_000717.2 | CA4 | 762 | 10.84 | -5.35 | 3.89 | -2.82 | 4.36E-04 |
| ILMN_10025 | NM_002934.2 | RNASE2 | 6036 | 8.72 | -5.33 | 3.85 | -1.66 | 4.43E-04 |
| ILMN_21254 | NM_024788.2 | FLJ21062 | 79846 | 9.97 | 5.32 | 3.82 | 1.10 | 4.50E-04 |
| ILMN_9361 | NM_199127.1 | GGTL4 | 91227 | 8.36 | -5.32 | 3.82 | -1.11 | 4.50E-04 |
| ILMN_14294 | NM_175892.3 | FLJ37266 | 283225 | 8.81 | 5.31 | 3.79 | 1.00 | 4.59E-04 |
| ILMN_21047 | NM_172313.1 | CSF3R | 1441 | 11.05 | -5.30 | 3.76 | -1.05 | 4.67E-04 |
| ILMN_28694 | NM_001792.2 | CDH2 | 1000 | 9.21 | 5.26 | 3.63 | 1.53 | 5.17E-04 |
| ILMN_9515 | NM_005460.2 | SNCAIP | 9627 | 9.34 | 5.23 | 3.56 | 1.05 | 5.43E-04 |
| ILMN_12987 | NM_000493.2 | COL10A1 | 1300 | 8.11 | 5.23 | 3.54 | 1.31 | 5.53E-04 |
| ILMN_6151 | NM_003248.3 | THBS4 | 7060 | 8.75 | 5.22 | 3.52 | 1.15 | 5.58E-04 |
| ILMN_2022 | NM_000024.3 | ADRB2 | 154 | 10.83 | -5.21 | 3.48 | -1.12 | 5.78E-04 |
| ILMN_28723 | NM_013230.2 | CD24 | 934 | 13.17 | 5.20 | 3.47 | 1.45 | 5.80E-04 |
| ILMN_24098 | NM_144962.1 | PEBP4 | 157310 | 10.34 | -5.20 | 3.45 | -1.94 | 5.87E-04 |
| ILMN_3875 | NM_001078.2 | VCAM1 | 7412 | 11.48 | 5.19 | 3.44 | 2.36 | 5.88E-04 |
| ILMN_10629 | NM_000679.3 | ADRA1B | 147 | 8.64 | -5.18 | 3.41 | -1.26 | 6.01E-04 |
| ILMN_16682 | NM_005585.2 | SMAD6 | 4091 | 11.07 | -5.15 | 3.32 | -1.85 | 6.49E-04 |
| ILMN_15768 | NM_017434.3 | DUOX1 | 53905 | 10.46 | -5.14 | 3.29 | -1.09 | 6.63E-04 |
| ILMN_5494 | NM_014638.2 | PLCH2 | 9651 | 9.93 | 5.14 | 3.28 | 1.71 | 6.70E-04 |
| ILMN_6449 | NM_001004019.1 | FBLN2 | 2199 | 11.20 | 5.13 | 3.27 | 1.06 | 6.74E-04 |
| ILMN_17159 | NM_000174.2 | GP9 | 2815 | 7.53 | -5.12 | 3.24 | -1.05 | 6.89E-04 |
| ILMN_16684 | NM_031950.2 | KSP37 | 83888 | 8.10 | -5.11 | 3.21 | -1.32 | 7.04E-04 |
| ILMN_22078 | NM_003803.2 | MYOM1 | 8736 | 9.69 | 5.07 | 3.09 | 1.08 | 7.62E-04 |
| ILMN_17741 | NM_032883.1 | C20ORF100 | 84969 | 12.39 | -5.05 | 3.03 | -1.14 | 8.02E-04 |
| ILMN_19246 | NM_016610.2 | TLR8 | 51311 | 8.98 | -5.03 | 2.99 | -1.10 | 8.32E-04 |
| ILMN_15660 | NM_014839.3 | LPPR4 | 9890 | 8.46 | 5.03 | 2.96 | 1.38 | 8.38E-04 |
| ILMN_5692 | NM_000020.1 | ACVRL1 | 94 | 10.67 | -5.01 | 2.93 | -1.06 | 8.56E-04 |
| ILMN_19892 | NM_016413.3 | CPB2 | 1361 | 7.96 | -5.01 | 2.93 | -1.23 | 8.56E-04 |
| ILMN_27029 | NM_004364.2 | CEBPA | 1050 | 11.15 | -5.00 | 2.89 | -1.25 | 8.85E-04 |
| ILMN_11539 | NM_153283.1 | HYAL1 | 3373 | 10.57 | -4.96 | 2.78 | -1.10 | 9.61E-04 |
| ILMN_6004 | NM_001033886.1 | CXCL12 | 6387 | 11.32 | 4.94 | 2.71 | 1.03 | 1.01E-03 |
| ILMN_550 | NM_001001994.1 | GPM6B | 2824 | 9.57 | -4.94 | 2.72 | -1.10 | 1.01E-03 |
| ILMN_14950 | NM_002251.3 | KCNS1 | 3787 | 7.95 | -4.94 | 2.71 | -1.16 | 1.01E-03 |
| ILMN_3962 | NM_016509.2 | CLEC1B | 51266 | 7.53 | -4.91 | 2.62 | -1.40 | 1.08E-03 |
| ILMN_21430 | NM_152611.2 | C20ORF75 | 164312 | 8.91 | -4.88 | 2.55 | -1.09 | 1.14E-03 |
| ILMN_25431 | NM_005063.4 | SCD | 6319 | 13.11 | -4.88 | 2.54 | -1.23 | 1.15E-03 |
| ILMN_15847 | NM_203418.1 | DSCR1 | 1827 | 10.45 | 4.87 | 2.53 | 1.01 | 1.15E-03 |
| ILMN_17706 | NM_001406.3 | EFNB3 | 1949 | 8.83 | 4.84 | 2.45 | 1.08 | 1.22E-03 |
| ILMN_25950 | NM_014767.1 | SPOCK2 | 9806 | 12.34 | -4.82 | 2.38 | -1.75 | 1.28E-03 |
| ILMN_8351 | NM_002837.2 | PTPRB | 5787 | 10.53 | -4.79 | 2.30 | -1.20 | 1.36E-03 |
| ILMN_29001 | NM_000928.2 | PLA2G1B | 5319 | 9.80 | -4.79 | 2.29 | -2.21 | 1.37E-03 |
| ILMN_12248 | NM_001710.4 | CFB | 629 | 12.56 | 4.76 | 2.22 | 1.06 | 1.45E-03 |
| ILMN_14249 | NM_198148.1 | CPXM2 | 119587 | 10.44 | 4.75 | 2.18 | 1.39 | 1.50E-03 |
| ILMN_26947 | NM_181882.2 | PRX | 57716 | 8.28 | -4.72 | 2.09 | -1.18 | 1.61E-03 |
| ILMN_23060 | NM_000089.3 | COL1A2 | 1278 | 12.60 | 4.71 | 2.08 | 1.68 | 1.63E-03 |
| ILMN_7811 | NM_205854.1 | SFTPG | 389376 | 12.53 | -4.71 | 2.07 | -1.41 | 1.63E-03 |
| ILMN_18898 | NM_152673.1 | MUC20 | 200958 | 9.62 | 4.70 | 2.04 | 1.08 | 1.67E-03 |
| ILMN_27281 | NM_004887.3 | CXCL14 | 9547 | 9.17 | 4.69 | 2.02 | 1.36 | 1.70E-03 |
| ILMN_18675 | NM_152717.1 | MGC35295 | 219995 | 8.25 | -4.69 | 2.00 | -1.30 | 1.73E-03 |
| ILMN_24839 | NM_138370.1 | LOC91461 | 91461 | 9.75 | -4.62 | 1.82 | -1.01 | 1.99E-03 |
| ILMN_5478 | NM_006574.2 | CSPG5 | 10675 | 8.37 | -4.62 | 1.82 | -1.04 | 1.99E-03 |
| ILMN_176190 | NM_001040114.1 | MYH11 | 4629 | 13.87 | 4.61 | 1.79 | 1.05 | 2.03E-03 |
| ILMN_21715 | NM_014398.2 | LAMP3 | 27074 | 13.12 | -4.61 | 1.78 | -1.48 | 2.04E-03 |
| ILMN_13072 | NM_002964.3 | S100A8 | 6279 | 13.09 | -4.58 | 1.71 | -1.56 | 2.15E-03 |
| ILMN_18641 | NM_012258.2 | HEY1 | 23462 | 9.72 | -4.57 | 1.68 | -1.14 | 2.19E-03 |
| ILMN_22338 | NM_006843.2 | SDS | 10993 | 8.19 | 4.56 | 1.64 | 1.16 | 2.26E-03 |
| ILMN_20133 | NM_006398.2 | UBD | 10537 | 9.92 | 4.53 | 1.56 | 1.75 | 2.39E-03 |
| ILMN_174339 | NM_000115.1 | EDNRB | 1910 | 9.83 | -4.53 | 1.55 | -1.10 | 2.41E-03 |
| ILMN_11495 | NM_004751.1 | GCNT3 | 9245 | 8.42 | 4.51 | 1.51 | 1.04 | 2.49E-03 |
| ILMN_15617 | NM_003843.2 | SCEL | 8796 | 8.85 | -4.51 | 1.50 | -1.11 | 2.50E-03 |
| ILMN_16766 | NM_152520.3 | ZNF533 | 151126 | 10.54 | -4.51 | 1.49 | -1.62 | 2.52E-03 |
| ILMN_25646 | NM_002965.2 | S100A9 | 6280 | 13.26 | -4.50 | 1.48 | -1.50 | 2.54E-03 |
| ILMN_21318 | NM_152414.3 | BHLHB5 | 27319 | 9.52 | 4.49 | 1.44 | 1.18 | 2.62E-03 |
| ILMN_6723 | NM_173452.1 | FCN3 | 8547 | 13.52 | -4.47 | 1.39 | -2.08 | 2.71E-03 |
| ILMN_28129 | NM_000250.1 | MPO | 4353 | 7.76 | -4.46 | 1.37 | -1.01 | 2.75E-03 |
| ILMN_10476 | NM_022718.2 | MMP25 | 64386 | 8.52 | -4.46 | 1.37 | -1.05 | 2.75E-03 |
| ILMN_29396 | NM_005084.2 | PLA2G7 | 7941 | 10.57 | 4.46 | 1.35 | 1.41 | 2.79E-03 |
| ILMN_12373 | NM_001461.1 | FMO5 | 2330 | 9.42 | -4.45 | 1.33 | -1.53 | 2.82E-03 |
| ILMN_21799 | NM_001763.1 | CD1A | 909 | 8.86 | 4.45 | 1.32 | 1.52 | 2.83E-03 |
| ILMN_3636 | NM_003280.1 | TNNC1 | 7134 | 10.20 | -4.44 | 1.31 | -1.47 | 2.84E-03 |
| ILMN_4881 | NM_018286.1 | TMEM100 | 55273 | 11.35 | -4.44 | 1.30 | -2.16 | 2.86E-03 |
| ILMN_7706 | NM_017709.2 | FAM46C | 54855 | 11.83 | 4.42 | 1.25 | 1.16 | 2.99E-03 |
| ILMN_19677 | NM_004345.3 | CAMP | 820 | 8.78 | -4.41 | 1.21 | -1.15 | 3.07E-03 |
| ILMN_27565 | NM_004624.2 | VIPR1 | 7433 | 12.60 | -4.40 | 1.21 | -1.86 | 3.07E-03 |
| ILMN_13160 | NM_002639.2 | SERPINB5 | 5268 | 8.67 | 4.40 | 1.19 | 1.90 | 3.10E-03 |
| ILMN_29492 | NM_022963.2 | FGFR4 | 2264 | 9.49 | -4.39 | 1.17 | -1.08 | 3.13E-03 |
| ILMN_3809 | NM_148177.1 | FBXO32 | 114907 | 9.67 | 4.38 | 1.15 | 1.04 | 3.17E-03 |
| ILMN_8467 | NM_173843.1 | IL1RN | 3557 | 9.79 | 4.37 | 1.11 | 1.37 | 3.27E-03 |
| ILMN_17340 | NM_032532.1 | FNDC1 | 84624 | 8.29 | 4.36 | 1.08 | 1.16 | 3.35E-03 |
| ILMN_3436 | NM_002922.3 | RGS1 | 5996 | 11.75 | 4.36 | 1.08 | 1.70 | 3.35E-03 |
| ILMN_137320 | NM_004527.2 | MEOX1 | 4222 | 8.24 | 4.32 | 0.97 | 1.04 | 3.67E-03 |
| ILMN_11689 | NM_178012.3 | TUBB2B | 347733 | 9.27 | 4.30 | 0.93 | 1.23 | 3.77E-03 |
| ILMN_25079 | NM_025243.2 | SLC19A3 | 80704 | 8.76 | -4.30 | 0.92 | -1.06 | 3.79E-03 |
| ILMN_2381 | NM_000804.2 | FOLR3 | 2352 | 8.52 | -4.29 | 0.90 | -1.70 | 3.83E-03 |
| ILMN_13364 | NM_001615.3 | ACTG2 | 72 | 14.14 | 4.29 | 0.89 | 1.48 | 3.86E-03 |
| ILMN_16214 | NM_005940.3 | MMP11 | 4320 | 8.99 | 4.27 | 0.83 | 1.90 | 4.08E-03 |
| ILMN_16098 | NM_003247.2 | THBS2 | 7058 | 11.66 | 4.26 | 0.80 | 1.43 | 4.18E-03 |
| ILMN_16493 | NM_005358.3 | LMO7 | 4008 | 9.67 | -4.24 | 0.76 | -1.25 | 4.31E-03 |
| ILMN_8326 | NM_005342.2 | HMGB3 | 3149 | 9.66 | 4.23 | 0.73 | 1.20 | 4.43E-03 |
| ILMN_179575 | NM_000584.2 | IL8 | 3576 | 12.40 | 4.23 | 0.71 | 1.58 | 4.49E-03 |
| ILMN_8824 | NM_022910.1 | NDRG4 | 65009 | 9.17 | -4.21 | 0.67 | -1.36 | 4.63E-03 |
| ILMN_2315 | NM_006290.2 | TNFAIP3 | 7128 | 11.76 | 4.20 | 0.64 | 1.37 | 4.73E-03 |
| ILMN_14571 | NM_032034.1 | SLC4A11 | 83959 | 9.44 | 4.19 | 0.62 | 1.54 | 4.80E-03 |
| ILMN_19816 | NM_178232.2 | HAPLN3 | 145864 | 8.85 | 4.16 | 0.54 | 1.11 | 5.06E-03 |
| ILMN_18883 | NM_001608.2 | ACADL | 33 | 9.01 | -4.16 | 0.54 | -1.27 | 5.06E-03 |
| ILMN_38866 | NM_000459.2 | TEK | 7010 | 12.03 | -4.16 | 0.53 | -1.03 | 5.10E-03 |
| ILMN_23751 | NM_182536.2 | GDDR | 200504 | 12.10 | -4.14 | 0.49 | -1.59 | 5.27E-03 |
| ILMN_9861 | NM_002112.1 | HDC | 3067 | 10.88 | 4.14 | 0.48 | 1.56 | 5.28E-03 |
| ILMN_29392 | NM_001299.4 | CNN1 | 1264 | 10.94 | 4.13 | 0.45 | 1.28 | 5.42E-03 |
| ILMN_26128 | NM_000090.2 | COL3A1 | 1281 | 12.94 | 4.13 | 0.45 | 1.84 | 5.43E-03 |
| ILMN_16131 | NM_019102.2 | HOXA5 | 3202 | 12.09 | -4.11 | 0.40 | -1.04 | 5.62E-03 |
| ILMN_17459 | NM_006433.2 | GNLY | 10578 | 8.91 | -4.07 | 0.28 | -1.01 | 6.11E-03 |
| ILMN_25660 | NM_002036.2 | DARC | 2532 | 13.07 | 4.04 | 0.21 | 1.24 | 6.46E-03 |
| ILMN_12902 | NM_032793.2 | MFSD2 | 84879 | 9.42 | -4.04 | 0.21 | -1.01 | 6.46E-03 |
| ILMN_7654 | NM_000424.2 | KRT5 | 3852 | 9.02 | 4.04 | 0.20 | 2.26 | 6.47E-03 |
| ILMN_171409 | NM_003722.3 | TP73L | 8626 | 9.17 | 4.04 | 0.19 | 1.75 | 6.50E-03 |
| ILMN_7523 | NM_001322.2 | CST2 | 1470 | 8.03 | 4.03 | 0.19 | 1.17 | 6.50E-03 |
| ILMN_7546 | NM_001032360.1 | MMP19 | 4327 | 8.52 | 3.99 | 0.06 | 1.13 | 7.16E-03 |
| ILMN_25770 | NM_003894.3 | PER2 | 8864 | 10.00 | 3.96 | 0.00 | 1.05 | 7.54E-03 |
| ILMN_2606 | NM_014326.3 | DAPK2 | 23604 | 9.30 | -3.95 | -0.05 | -1.06 | 7.83E-03 |
| ILMN_11321 | NM_001138.1 | AGRP | 181 | 8.62 | -3.94 | -0.07 | -1.04 | 7.93E-03 |
| ILMN_7069 | NM_001002915.1 | IGFL2 | 147920 | 7.90 | 3.94 | -0.08 | 1.07 | 7.97E-03 |
| ILMN_27932 | NM_006875.2 | PIM2 | 11040 | 11.31 | 3.91 | -0.15 | 1.12 | 8.42E-03 |
| ILMN_25185 | NM_002982.3 | CCL2 | 6347 | 12.87 | 3.90 | -0.16 | 1.67 | 8.55E-03 |
| ILMN_26343 | NM_004004.3 | GJB2 | 2706 | 8.25 | 3.89 | -0.20 | 1.06 | 8.78E-03 |
| ILMN_2688 | NM_004864.1 | GDF15 | 9518 | 11.39 | 3.88 | -0.23 | 1.35 | 8.97E-03 |
| ILMN_23716 | NM_138461.2 | TM4SF19 | 116211 | 8.42 | 3.88 | -0.24 | 1.20 | 9.08E-03 |
| ILMN_179703 | NM_153366.2 | SVEP1 | 79987 | 12.26 | -3.87 | -0.26 | -1.05 | 9.18E-03 |
| ILMN_176524 | NM_000963.1 | PTGS2 | 5743 | 11.78 | 3.85 | -0.31 | 1.30 | 9.49E-03 |
| ILMN_23624 | NM_001453.1 | FOXC1 | 2296 | 11.72 | 3.84 | -0.32 | 1.21 | 9.60E-03 |
| ILMN_4650 | NM_022138.1 | SMOC2 | 64094 | 9.19 | 3.83 | -0.36 | 1.04 | 9.89E-03 |
| ILMN_13191 | NM_024697.1 | ZNF659 | 79750 | 9.34 | 3.81 | -0.40 | 1.28 | 1.02E-02 |
| ILMN_21913 | NM_017420.2 | SIX4 | 51804 | 9.46 | 3.82 | -0.40 | 1.27 | 1.02E-02 |
| ILMN_10372 | NM_006274.2 | CCL19 | 6363 | 10.30 | 3.79 | -0.46 | 1.71 | 1.06E-02 |
| ILMN_11342 | NM_024621.1 | VEPH1 | 79674 | 8.33 | -3.79 | -0.46 | -1.04 | 1.07E-02 |
| ILMN_19350 | NM_178452.3 | LRRC50 | 123872 | 10.43 | 3.77 | -0.53 | 1.85 | 1.12E-02 |
| ILMN_26004 | NM_213600.2 | PLA2G4F | 255189 | 10.11 | -3.77 | -0.53 | -1.40 | 1.12E-02 |
| ILMN_21263 | NM_030817.1 | APOLD1 | 81575 | 11.84 | 3.76 | -0.54 | 1.48 | 1.13E-02 |
| ILMN_20406 | NM_004669.2 | CLIC3 | 9022 | 10.29 | -3.75 | -0.57 | -1.09 | 1.15E-02 |
| ILMN_16948 | NM_030919.1 | C20ORF129 | 81610 | 9.25 | 3.74 | -0.61 | 1.08 | 1.19E-02 |
| ILMN_15943 | NM_005601.3 | NKG7 | 4818 | 10.40 | -3.72 | -0.66 | -1.12 | 1.23E-02 |
| ILMN_25604 | NM_144575.2 | CAPN13 | 92291 | 10.51 | 3.71 | -0.68 | 1.88 | 1.25E-02 |
| ILMN_137533 | NM_000393.2 | COL5A2 | 1290 | 11.72 | 3.63 | -0.89 | 1.13 | 1.46E-02 |
| ILMN_7147 | NM_001393.2 | ECM2 | 1842 | 10.05 | 3.63 | -0.89 | 1.09 | 1.46E-02 |
| ILMN_167992 | NM_003617.2 | RGS5 | 8490 | 9.06 | 3.60 | -0.97 | 1.12 | 1.55E-02 |
| ILMN_23377 | NM_033197.2 | C20ORF114 | 92747 | 13.08 | 3.60 | -0.97 | 3.56 | 1.56E-02 |
| ILMN_41107 | XM_939320.1 | LOC389816 | 389816 | 9.01 | 3.60 | -0.98 | 1.50 | 1.57E-02 |
| ILMN_27352 | NM_053277.1 | CLIC6 | 54102 | 12.41 | 3.59 | -0.99 | 1.30 | 1.58E-02 |
| ILMN_30234 | NM_003856.2 | IL1RL1 | 9173 | 10.26 | -3.59 | -1.01 | -2.15 | 1.60E-02 |
| ILMN_11306 | NM_005411.3 | SFTPA1 | 6435 | 11.01 | -3.58 | -1.03 | -1.40 | 1.63E-02 |
| ILMN_183137 | NM_001042507.1 | LOC653499 | 653499 | 8.69 | 3.56 | -1.07 | 1.98 | 1.67E-02 |
| ILMN_13668 | NM_015444.1 | RIS1 | 25907 | 10.32 | 3.56 | -1.09 | 1.02 | 1.69E-02 |
| ILMN_14586 | NM_002309.2 | LIF | 3976 | 8.26 | 3.55 | -1.09 | 1.00 | 1.70E-02 |
| ILMN_137665 | NM_018414.2 | ST6GALNAC1 | 55808 | 9.22 | 3.55 | -1.10 | 1.20 | 1.72E-02 |
| ILMN_181020 | NM_003890.1 | FCGBP | 8857 | 10.74 | 3.55 | -1.11 | 1.28 | 1.73E-02 |
| ILMN_2613 | NM_004962.2 | GDF10 | 2662 | 9.54 | -3.53 | -1.15 | -1.20 | 1.78E-02 |
| ILMN_8954 | NM_001072.2 | UGT1A6 | 54578 | 8.08 | 3.53 | -1.16 | 1.14 | 1.79E-02 |
| ILMN_17948 | NM_001927.3 | DES | 1674 | 10.65 | 3.50 | -1.23 | 1.27 | 1.90E-02 |
| ILMN_14283 | NM_080617.4 | CBLN4 | 140689 | 8.29 | 3.50 | -1.23 | 1.18 | 1.90E-02 |
| ILMN_25628 | NM_173515.2 | CNKSR3 | 154043 | 10.76 | 3.50 | -1.23 | 1.03 | 1.90E-02 |
| ILMN_29440 | NM_024626.1 | VTCN1 | 79679 | 8.41 | 3.47 | -1.30 | 1.46 | 1.98E-02 |
| ILMN_15989 | NM_001005505.1 | CACNA2D2 | 9254 | 10.27 | -3.46 | -1.33 | -1.12 | 2.03E-02 |
| ILMN_8268 | NM_025135.2 | FHOD3 | 80206 | 8.87 | 3.46 | -1.34 | 1.09 | 2.04E-02 |
| ILMN_28510 | NM_025251.1 | KIAA1688 | 80728 | 9.49 | 3.43 | -1.40 | 1.02 | 2.14E-02 |
| ILMN_30351 | NM_002307.1 | LGALS7 | 3963 | 8.67 | 3.43 | -1.41 | 1.66 | 2.16E-02 |
| ILMN_6391 | NM_001458.2 | FLNC | 2318 | 9.81 | 3.42 | -1.42 | 1.31 | 2.18E-02 |
| ILMN_6390 | NM_000691.3 | ALDH3A1 | 218 | 9.85 | 3.42 | -1.44 | 1.63 | 2.20E-02 |
| ILMN_10469 | NM_006198.2 | PCP4 | 5121 | 8.22 | 3.37 | -1.55 | 1.42 | 2.38E-02 |
| ILMN_3189 | NM_002275.2 | KRT15 | 3866 | 10.21 | 3.36 | -1.57 | 1.95 | 2.42E-02 |
| ILMN_22279 | NM_015507.2 | EGFL6 | 25975 | 10.08 | 3.35 | -1.61 | 1.16 | 2.48E-02 |
| ILMN_9752 | NM_006820.1 | IFI44L | 10964 | 10.53 | 3.35 | -1.61 | 1.03 | 2.50E-02 |
| ILMN_8057 | NM_003956.2 | CH25H | 9023 | 11.17 | 3.33 | -1.66 | 1.11 | 2.58E-02 |
| ILMN_19608 | NM_001002026.2 | CLDN18 | 51208 | 11.91 | -3.31 | -1.70 | -1.84 | 2.66E-02 |
| ILMN_30031 | NM_145740.2 | GSTA1 | 2938 | 10.08 | 3.30 | -1.74 | 1.82 | 2.73E-02 |
| ILMN_22396 | NM_002630.1 | PGC | 5225 | 14.13 | -3.29 | -1.75 | -1.05 | 2.75E-02 |
| ILMN_14456 | NM_000185.3 | SERPIND1 | 3053 | 8.47 | 3.28 | -1.79 | 1.40 | 2.85E-02 |
| ILMN_24314 | NM_004024.3 | ATF3 | 467 | 10.15 | 3.27 | -1.80 | 1.02 | 2.85E-02 |
| ILMN_20280 | NM_145263.1 | SPATA18 | 132671 | 10.73 | 3.27 | -1.81 | 1.35 | 2.87E-02 |
| ILMN_12288 | NM_020879.1 | KIAA1505 | 57639 | 9.97 | 3.27 | -1.82 | 1.20 | 2.89E-02 |
| ILMN_22857 | NM_001001437.3 | CCL3L3 | 414062 | 11.05 | 3.25 | -1.86 | 1.32 | 2.98E-02 |
| ILMN_6469 | NM_000600.1 | IL6 | 3569 | 11.80 | 3.24 | -1.89 | 1.61 | 3.05E-02 |
| ILMN_10556 | NM_018593.3 | SLC16A10 | 117247 | 9.81 | 3.17 | -2.05 | 1.04 | 3.43E-02 |
| ILMN_19978 | NM_006235.1 | POU2AF1 | 5450 | 8.83 | 3.16 | -2.08 | 1.04 | 3.52E-02 |
| ILMN_3183 | NM_016354.3 | SLCO4A1 | 28231 | 10.31 | -3.14 | -2.13 | -1.22 | 3.64E-02 |
| ILMN_20817 | NM_021615.3 | CHST6 | 4166 | 8.73 | 3.12 | -2.18 | 1.14 | 3.81E-02 |
| ILMN_21827 | NM_006017.1 | PROM1 | 8842 | 10.85 | 3.10 | -2.22 | 2.12 | 3.93E-02 |
| ILMN_168618 | NM_007036.3 | ESM1 | 11082 | 8.73 | -3.07 | -2.29 | -1.48 | 4.12E-02 |
| ILMN_12444 | NM_001023565.1 | LOC401286 | 401286 | 9.17 | -3.02 | -2.43 | -1.33 | 4.57E-02 |
| ILMN_7166 | NM_002193.1 | INHBB | 3625 | 9.22 | 3.00 | -2.46 | 1.05 | 4.64E-02 |
| ILMN_20473 | NM_005554.3 | KRT6A | 3853 | 9.64 | 2.97 | -2.53 | 2.30 | 4.91E-02 |
| ILMN_15389 | NM_007281.1 | SCRG1 | 11341 | 9.02 | 2.97 | -2.55 | 1.05 | 4.96E-02 |
| ILMN_9269 | NM_024889.3 | C10ORF81 | 79949 | 9.23 | 2.95 | -2.58 | 1.64 | 5.07E-02 |
| ILMN_169048 | NM_006926.1 | SFTPA2 | 6436 | 12.14 | -2.94 | -2.60 | -1.56 | 5.16E-02 |
| ILMN_4442 | NM_002425.1 | MMP10 | 4319 | 8.43 | 2.92 | -2.65 | 1.31 | 5.34E-02 |
| ILMN_23096 | NM_052863.2 | SCGB3A1 | 92304 | 14.54 | 2.90 | -2.70 | 1.66 | 5.55E-02 |
| ILMN_8593 | NM_001337.3 | CX3CR1 | 1524 | 8.97 | -2.89 | -2.72 | -1.09 | 5.63E-02 |
| ILMN_25404 | NM_003122.2 | SPINK1 | 6690 | 8.68 | 2.83 | -2.85 | 1.07 | 6.19E-02 |
| ILMN_15634 | NM_005623.2 | CCL8 | 6355 | 10.42 | 2.83 | -2.86 | 1.07 | 6.23E-02 |
| ILMN_4340 | NM_020070.2 | IGLL1 | 3543 | 13.31 | 2.82 | -2.89 | 1.09 | 6.36E-02 |
| ILMN_22042 | NM_001045.2 | SLC6A4 | 6532 | 9.45 | -2.79 | -2.96 | -1.75 | 6.71E-02 |
| ILMN_12592 | NM_206833.1 | CTXN1 | 404217 | 10.72 | 2.77 | -2.99 | 1.21 | 6.88E-02 |
| ILMN_7052 | NM_005727.2 | TSPAN1 | 10103 | 10.18 | 2.77 | -3.00 | 1.38 | 6.92E-02 |
| ILMN_18800 | NM_001089.1 | ABCA3 | 21 | 11.34 | -2.75 | -3.04 | -1.01 | 7.13E-02 |
| ILMN_16264 | NM_031422.1 | CHST9 | 83539 | 9.09 | 2.75 | -3.05 | 1.40 | 7.17E-02 |
| ILMN_14080 | NM_001898.2 | CST1 | 1469 | 7.80 | 2.71 | -3.13 | 1.07 | 7.61E-02 |
| ILMN_26176 | NM_021870.2 | FGG | 2266 | 8.68 | -2.71 | -3.13 | -1.50 | 7.66E-02 |
| ILMN_20794 | NM_005564.2 | LCN2 | 3934 | 12.51 | 2.68 | -3.20 | 1.46 | 8.09E-02 |
| ILMN_8636 | NM_016459.3 | MGC29506 | 51237 | 11.16 | 2.63 | -3.31 | 1.25 | 8.73E-02 |
| ILMN_18387 | NM_014178.6 | STXBP6 | 29091 | 9.01 | -2.63 | -3.31 | -1.04 | 8.73E-02 |
| ILMN_7438 | NM_003645.2 | SLC27A2 | 11001 | 9.38 | 2.62 | -3.34 | 1.01 | 8.95E-02 |
| ILMN_22283 | NM_003063.1 | SLN | 6588 | 9.93 | 2.58 | -3.41 | 1.08 | 9.42E-02 |
| ILMN_5066 | NM_001565.1 | CXCL10 | 3627 | 11.16 | 2.58 | -3.41 | 2.02 | 9.43E-02 |
| ILMN_11739 | NM_002198.1 | IRF1 | 3659 | 11.95 | 2.55 | -3.48 | 1.05 | 9.90E-02 |
