# Supplementary material for: Prominence of IL6, IGF, TLR, and Bioenergetics Pathway Perturbation in Lung Tissues of Scleroderma Patients With Pulmonary Fibrosis
Source: Front Immunol. 2020 Mar 10;11:383. doi: 10.3389/fimmu.2020.00383 (PMC7075854; doi:10.3389/fimmu.2020.00383)
Supplement: Supplementary file 2 [file Table_2.DOCX]

***Supplementary Table 2***

Prominence of IL6, IGF, TLR and bioenergetics pathway perturbation in lung tissues of scleroderma patients with pulmonary fibrosis

**Ludivine Renaud****^1^, Willian A. da Silveira^2^, Naoko Takamura^1^, Gary Hardiman^2^, Carol Feghali-Bostwick^1^***

^1^ Department of Medicine, Medical University of South Carolina, Charleston, SC, USA.

^2^ School of Biological Sciences and Institute for Global Food Security, Queens University Belfast, Belfast BT9 5AG, UK.

*** Correspondence:**Dr. Carol Feghali-Bostwick
feghalib@musc.edu

**Supplementary Table 2:** **List of DE genes in IPF tissue**. The criteria for significance are q<0.1, logFC>1: upregulated (in red), logFC<-1: downregulated (in blue). Data is sorted on q-value from smallest to highest.

| **ID_REF** | **NCBI_Reference** | **HUGO_Symbol** | **Entrez_ID** | **AveExpr** | **t** | **B** | **logFC** | **q-value** |
| --- | --- | --- | --- | --- | --- | --- | --- | --- |
| ILMN_4766 | NM_002391.3 | MDK | 4192 | 9.73 | 10.74 | 18.15 | 1.57 | 3.05E-08 |
| ILMN_27702 | NM_053279.1 | C8ORF13 | 83648 | 8.88 | -10.06 | 16.55 | -1.45 | 8.58E-08 |
| ILMN_5174 | NM_173485.2 | ZNF218 | 128553 | 10.56 | 9.72 | 15.74 | 1.33 | 1.36E-07 |
| ILMN_12837 | NM_006579.1 | EBP | 10682 | 10.32 | -9.21 | 14.48 | -1.13 | 3.97E-07 |
| ILMN_11006 | NM_004525.1 | LRP2 | 4036 | 7.86 | -8.85 | 13.56 | -1.06 | 6.03E-07 |
| ILMN_20021 | NM_032638.3 | GATA2 | 2624 | 9.57 | -8.89 | 13.68 | -1.83 | 6.03E-07 |
| ILMN_27777 | NM_014573.1 | TMEM97 | 27346 | 10.85 | -8.71 | 13.21 | -1.48 | 7.65E-07 |
| ILMN_21257 | NM_021158.3 | TRIB3 | 57761 | 9.67 | -8.65 | 13.06 | -1.32 | 8.01E-07 |
| ILMN_137905 | NM_002084.2 | GPX3 | 2878 | 14.41 | -8.60 | 12.93 | -1.32 | 8.24E-07 |
| ILMN_24078 | NM_003013.2 | SFRP2 | 6423 | 11.42 | 8.49 | 12.63 | 2.64 | 1.03E-06 |
| ILMN_2390 | NM_023930.2 | KCTD14 | 65987 | 9.78 | -8.26 | 12.03 | -1.40 | 1.65E-06 |
| ILMN_21050 | NM_001007023.1 | DIO2 | 1734 | 8.67 | 8.23 | 11.96 | 1.58 | 1.66E-06 |
| ILMN_139058 | NM_005161.2 | AGTRL1 | 187 | 9.74 | 8.14 | 11.70 | 2.59 | 1.83E-06 |
| ILMN_4355 | NM_024579.1 | C1ORF54 | 79630 | 11.78 | 8.09 | 11.57 | 1.36 | 1.83E-06 |
| ILMN_138379 | NM_004055.3 | CAPN5 | 726 | 9.92 | 8.13 | 11.69 | 1.17 | 1.83E-06 |
| ILMN_9651 | NM_014452.3 | TNFRSF21 | 27242 | 12.73 | 8.09 | 11.57 | 1.09 | 1.83E-06 |
| ILMN_22736 | NM_006288.2 | THY1 | 7070 | 11.30 | 8.02 | 11.39 | 2.54 | 2.11E-06 |
| ILMN_975 | NM_018945.3 | PDE7B | 27115 | 9.55 | 7.79 | 10.77 | 1.07 | 3.12E-06 |
| ILMN_18738 | NM_013402.3 | FADS1 | 3992 | 10.22 | -7.79 | 10.78 | -1.23 | 3.12E-06 |
| ILMN_22426 | NM_013279.1 | C11ORF9 | 745 | 8.11 | -7.80 | 10.80 | -1.44 | 3.12E-06 |
| ILMN_176067 | NM_001042500.1 | LOC653600 | 728358 | 8.23 | -7.84 | 10.92 | -2.37 | 3.12E-06 |
| ILMN_16923 | NM_000396.2 | CTSK | 1513 | 11.26 | 7.68 | 10.48 | 1.62 | 3.82E-06 |
| ILMN_26801 | NM_020724.1 | RNF150 | 57484 | 9.65 | 7.68 | 10.49 | 1.11 | 3.82E-06 |
| ILMN_16128 | NM_004104.4 | FASN | 2194 | 12.78 | -7.68 | 10.48 | -1.68 | 3.82E-06 |
| ILMN_12246 | NM_001001438.1 | LSS | 4047 | 9.97 | -7.61 | 10.30 | -1.02 | 4.31E-06 |
| ILMN_26352 | NM_003468.2 | FZD5 | 7855 | 8.36 | -7.48 | 9.94 | -1.08 | 5.78E-06 |
| ILMN_12740 | NM_001793.3 | CDH3 | 1001 | 10.18 | 7.39 | 9.67 | 1.84 | 7.19E-06 |
| ILMN_20831 | NM_001450.3 | FHL2 | 2274 | 10.42 | 7.33 | 9.53 | 1.61 | 7.93E-06 |
| ILMN_4697 | NM_014696.2 | KIAA0514 | 9721 | 9.41 | -7.34 | 9.55 | -1.44 | 7.93E-06 |
| ILMN_5749 | NM_006163.1 | NFE2 | 4778 | 9.16 | -7.29 | 9.40 | -1.96 | 8.56E-06 |
| ILMN_2640 | NM_022351.2 | EFCBP1 | 64168 | 7.43 | -7.25 | 9.30 | -1.04 | 9.34E-06 |
| ILMN_28163 | NM_000758.2 | CSF2 | 1437 | 7.92 | -7.14 | 8.99 | -1.20 | 1.15E-05 |
| ILMN_17437 | NM_016286.2 | DCXR | 51181 | 11.40 | -7.13 | 8.96 | -1.04 | 1.16E-05 |
| ILMN_26681 | NM_030926.4 | ITM2C | 81618 | 11.69 | 7.07 | 8.80 | 1.09 | 1.35E-05 |
| ILMN_5522 | NM_001031692.1 | LRRC17 | 10234 | 8.66 | 7.00 | 8.61 | 1.33 | 1.55E-05 |
| ILMN_163190 | NM_015993.1 | TM4SF11 | 51090 | 10.46 | -6.91 | 8.35 | -1.78 | 1.91E-05 |
| ILMN_12517 | NM_022746.2 | MOSC1 | 64757 | 9.95 | -6.85 | 8.19 | -1.19 | 2.22E-05 |
| ILMN_20095 | NM_206930.1 | SYTL2 | 54843 | 9.91 | 6.83 | 8.13 | 1.03 | 2.29E-05 |
| ILMN_24828 | NM_001852.3 | COL9A2 | 1298 | 9.29 | 6.77 | 7.96 | 1.10 | 2.60E-05 |
| ILMN_11220 | NM_005217.2 | DEFA3 | 1668 | 8.63 | -6.78 | 7.97 | -2.51 | 2.60E-05 |
| ILMN_6950 | NM_003020.1 | SCG5 | 6447 | 8.93 | 6.73 | 7.84 | 1.36 | 2.85E-05 |
| ILMN_18980 | NM_002130.4 | HMGCS1 | 3157 | 11.69 | -6.72 | 7.83 | -1.26 | 2.85E-05 |
| ILMN_16252 | NM_001878.2 | CRABP2 | 1382 | 9.02 | 6.66 | 7.65 | 1.42 | 3.24E-05 |
| ILMN_29922 | NM_015419.1 | MXRA5 | 25878 | 11.86 | 6.62 | 7.53 | 1.67 | 3.60E-05 |
| ILMN_5566 | NM_000422.1 | KRT17 | 3872 | 11.13 | 6.61 | 7.50 | 3.22 | 3.66E-05 |
| ILMN_11215 | NM_002686.2 | PNMT | 5409 | 7.77 | -6.57 | 7.39 | -1.21 | 3.94E-05 |
| ILMN_20370 | NM_005261.2 | GEM | 2669 | 9.49 | 6.52 | 7.26 | 1.23 | 4.39E-05 |
| ILMN_9853 | NR_002766.1 | MEG3 | 55384 | 10.80 | 6.50 | 7.19 | 1.34 | 4.54E-05 |
| ILMN_11548 | NM_000088.2 | COL1A1 | 1277 | 12.84 | 6.47 | 7.11 | 2.29 | 4.71E-05 |
| ILMN_1899 | NM_139212.2 | HOP | 84525 | 12.17 | -6.46 | 7.09 | -1.36 | 4.74E-05 |
| ILMN_17467 | NM_180991.4 | SLCO4C1 | 353189 | 8.53 | -6.40 | 6.92 | -1.33 | 5.47E-05 |
| ILMN_27165 | NM_015715.2 | PLA2G3 | 50487 | 9.03 | -6.41 | 6.93 | -1.41 | 5.47E-05 |
| ILMN_11566 | NM_000240.2 | MAOA | 4128 | 13.46 | -6.37 | 6.82 | -1.02 | 5.98E-05 |
| ILMN_19248 | NM_000954.5 | PTGDS | 5730 | 13.03 | 6.33 | 6.72 | 1.34 | 6.28E-05 |
| ILMN_12251 | NM_001006932.1 | RPS6KA2 | 6196 | 11.00 | -6.34 | 6.73 | -1.08 | 6.28E-05 |
| ILMN_18400 | NM_001925.1 | DEFA4 | 1669 | 7.78 | -6.27 | 6.55 | -1.03 | 7.09E-05 |
| ILMN_1036 | NM_004000.2 | CHI3L2 | 1117 | 9.40 | -6.26 | 6.51 | -1.68 | 7.24E-05 |
| ILMN_29692 | NM_004084.2 | DEFA1 | 1667 | 9.22 | -6.24 | 6.46 | -2.80 | 7.47E-05 |
| ILMN_16948 | NM_030919.1 | C20ORF129 | 81610 | 9.25 | 6.23 | 6.44 | 1.80 | 7.56E-05 |
| ILMN_15885 | NM_001311.3 | CRIP1 | 1396 | 13.56 | 6.23 | 6.41 | 1.16 | 7.59E-05 |
| ILMN_12582 | NM_134421.1 | HPCAL1 | 3241 | 11.57 | -6.23 | 6.42 | -1.01 | 7.59E-05 |
| ILMN_828 | NM_080878.2 | ITLN2 | 142683 | 8.92 | -6.18 | 6.27 | -2.67 | 8.62E-05 |
| ILMN_15049 | NM_000095.2 | COMP | 1311 | 10.88 | 6.14 | 6.18 | 2.46 | 8.96E-05 |
| ILMN_11594 | NM_012261.2 | C20ORF103 | 24141 | 8.91 | 6.15 | 6.19 | 1.52 | 8.96E-05 |
| ILMN_28977 | NM_014890.1 | DOC1 | 11259 | 11.28 | 6.14 | 6.18 | 1.08 | 8.96E-05 |
| ILMN_27995 | NM_018058.4 | CRTAC1 | 55118 | 10.39 | -6.15 | 6.20 | -2.13 | 8.96E-05 |
| ILMN_138322 | NM_007350.2 | PHLDA1 | 22822 | 12.12 | 6.09 | 6.01 | 1.48 | 1.06E-04 |
| ILMN_11485 | NM_177403.3 | RAB7B | 338382 | 10.14 | 6.01 | 5.79 | 1.27 | 1.26E-04 |
| ILMN_20087 | NM_000494.2 | COL17A1 | 1308 | 8.94 | 5.99 | 5.73 | 1.94 | 1.31E-04 |
| ILMN_6449 | NM_001004019.1 | FBLN2 | 2199 | 11.20 | 5.97 | 5.67 | 1.23 | 1.37E-04 |
| ILMN_28723 | NM_013230.2 | CD24 | 934 | 13.17 | 5.94 | 5.59 | 1.65 | 1.49E-04 |
| ILMN_26240 | NM_001257.3 | CDH13 | 1012 | 8.17 | -5.91 | 5.52 | -1.02 | 1.54E-04 |
| ILMN_24830 | NM_000094.2 | COL7A1 | 1294 | 10.93 | 5.90 | 5.49 | 1.92 | 1.56E-04 |
| ILMN_16950 | NM_024728.1 | C7ORF10 | 79783 | 8.67 | 5.90 | 5.47 | 1.18 | 1.59E-04 |
| ILMN_6746 | NM_002988.2 | CCL18 | 6362 | 9.32 | 5.87 | 5.38 | 1.42 | 1.71E-04 |
| ILMN_21047 | NM_172313.1 | CSF3R | 1441 | 11.05 | -5.81 | 5.22 | -1.15 | 1.92E-04 |
| ILMN_16792 | NM_021935.2 | PROK2 | 60675 | 8.94 | -5.80 | 5.20 | -2.13 | 1.92E-04 |
| ILMN_13811 | NM_003239.1 | TGFB3 | 7043 | 10.77 | 5.78 | 5.15 | 1.22 | 1.98E-04 |
| ILMN_6004 | NM_001033886.1 | CXCL12 | 6387 | 11.32 | 5.78 | 5.12 | 1.20 | 2.00E-04 |
| ILMN_21395 | NM_002990.3 | CCL22 | 6367 | 8.85 | 5.77 | 5.10 | 1.49 | 2.04E-04 |
| ILMN_2732 | NM_000597.2 | IGFBP2 | 3485 | 11.58 | 5.71 | 4.94 | 1.27 | 2.30E-04 |
| ILMN_12902 | NM_032793.2 | MFSD2 | 84879 | 9.42 | -5.71 | 4.94 | -1.43 | 2.30E-04 |
| ILMN_138827 | NM_145810.1 | CDCA7 | 83879 | 8.54 | 5.71 | 4.93 | 1.10 | 2.31E-04 |
| ILMN_17340 | NM_032532.1 | FNDC1 | 84624 | 8.29 | 5.67 | 4.82 | 1.51 | 2.47E-04 |
| ILMN_27781 | NM_014476.1 | PDLIM3 | 27295 | 11.87 | 5.64 | 4.74 | 1.16 | 2.64E-04 |
| ILMN_2442 | NM_004772.1 | C5ORF13 | 9315 | 10.91 | 5.64 | 4.72 | 1.30 | 2.68E-04 |
| ILMN_11545 | NM_001136.3 | AGER | 177 | 10.24 | -5.63 | 4.72 | -2.30 | 2.68E-04 |
| ILMN_15768 | NM_017434.3 | DUOX1 | 53905 | 10.46 | -5.63 | 4.71 | -1.20 | 2.70E-04 |
| ILMN_5029 | NM_000071.1 | CBS | 875 | 9.54 | -5.58 | 4.57 | -1.25 | 3.00E-04 |
| ILMN_170763 | NM_001042459.1 | FILIP1L | 11259 | 11.46 | 5.57 | 4.54 | 1.05 | 3.04E-04 |
| ILMN_9188 | NM_002423.3 | MMP7 | 4316 | 12.41 | 5.56 | 4.50 | 3.10 | 3.15E-04 |
| ILMN_3436 | NM_002922.3 | RGS1 | 5996 | 11.75 | 5.50 | 4.35 | 2.15 | 3.49E-04 |
| ILMN_20214 | NM_000640.2 | IL13RA2 | 3598 | 8.47 | 5.50 | 4.33 | 1.43 | 3.49E-04 |
| ILMN_13049 | NM_001001991.1 | DKFZP586H2123 | 25891 | 9.45 | 5.50 | 4.33 | 1.26 | 3.49E-04 |
| ILMN_13709 | NM_145764.1 | MGST1 | 4257 | 11.12 | -5.50 | 4.33 | -1.07 | 3.49E-04 |
| ILMN_27823 | NM_001029999.1 | MGC26718 | 440482 | 8.59 | 5.49 | 4.30 | 1.11 | 3.58E-04 |
| ILMN_26228 | NM_003358.1 | UGCG | 7357 | 10.93 | 5.47 | 4.25 | 1.01 | 3.69E-04 |
| ILMN_10970 | NM_001900.4 | CST5 | 1473 | 8.17 | 5.44 | 4.17 | 1.05 | 3.96E-04 |
| ILMN_26397 | NM_003725.2 | HSD17B6 | 8630 | 10.52 | -5.44 | 4.17 | -1.89 | 3.96E-04 |
| ILMN_27413 | NM_144707.1 | PROM2 | 150696 | 10.07 | 5.43 | 4.13 | 2.01 | 4.02E-04 |
| ILMN_5494 | NM_014638.2 | PLCH2 | 9651 | 9.93 | 5.44 | 4.15 | 1.82 | 4.02E-04 |
| ILMN_25111 | NM_000693.1 | ALDH1A3 | 220 | 11.35 | 5.43 | 4.14 | 1.60 | 4.02E-04 |
| ILMN_28982 | NM_015170.1 | SULF1 | 23213 | 10.72 | 5.43 | 4.13 | 1.34 | 4.02E-04 |
| ILMN_10025 | NM_002934.2 | RNASE2 | 6036 | 8.72 | -5.43 | 4.13 | -1.69 | 4.02E-04 |
| ILMN_18005 | NM_033393.1 | KIAA1727 | 85462 | 9.86 | -5.43 | 4.12 | -1.12 | 4.07E-04 |
| ILMN_12373 | NM_001461.1 | FMO5 | 2330 | 9.42 | -5.42 | 4.10 | -1.87 | 4.09E-04 |
| ILMN_24098 | NM_144962.1 | PEBP4 | 157310 | 10.34 | -5.41 | 4.08 | -2.02 | 4.18E-04 |
| ILMN_14815 | NM_004170.4 | SLC1A1 | 6505 | 8.63 | -5.40 | 4.05 | -1.15 | 4.24E-04 |
| ILMN_16766 | NM_152520.3 | ZNF533 | 151126 | 10.54 | -5.38 | 4.00 | -1.94 | 4.42E-04 |
| ILMN_28002 | NM_181353.1 | ID1 | 3397 | 11.80 | -5.38 | 3.98 | -1.17 | 4.45E-04 |
| ILMN_4927 | NM_024508.3 | ZBED2 | 79413 | 8.48 | -5.35 | 3.91 | -1.57 | 4.65E-04 |
| ILMN_25235 | NM_016206.2 | VGLL3 | 389136 | 9.37 | -5.32 | 3.81 | -1.11 | 5.12E-04 |
| ILMN_29001 | NM_000928.2 | PLA2G1B | 5319 | 9.80 | -5.25 | 3.61 | -2.43 | 5.92E-04 |
| ILMN_22078 | NM_003803.2 | MYOM1 | 8736 | 9.69 | 5.24 | 3.59 | 1.11 | 5.99E-04 |
| ILMN_9515 | NM_005460.2 | SNCAIP | 9627 | 9.34 | 5.21 | 3.49 | 1.05 | 6.35E-04 |
| ILMN_15732 | NM_001855.2 | COL15A1 | 1306 | 11.24 | 5.19 | 3.45 | 1.59 | 6.54E-04 |
| ILMN_17706 | NM_001406.3 | EFNB3 | 1949 | 8.83 | 5.19 | 3.43 | 1.16 | 6.58E-04 |
| ILMN_36330 | XM_941554.1 | KIAA1912 | 114800 | 8.90 | -5.19 | 3.43 | -1.22 | 6.58E-04 |
| ILMN_9394 | NM_000582.2 | SPP1 | 6696 | 11.30 | 5.13 | 3.28 | 3.18 | 7.35E-04 |
| ILMN_13364 | NM_001615.3 | ACTG2 | 72 | 14.14 | 5.13 | 3.26 | 1.77 | 7.41E-04 |
| ILMN_12928 | NM_004419.3 | DUSP5 | 1847 | 11.30 | 5.10 | 3.18 | 1.22 | 7.93E-04 |
| ILMN_11689 | NM_178012.3 | TUBB2B | 347733 | 9.27 | 5.09 | 3.16 | 1.45 | 8.08E-04 |
| ILMN_2315 | NM_006290.2 | TNFAIP3 | 7128 | 11.76 | 5.08 | 3.13 | 1.66 | 8.21E-04 |
| ILMN_10873 | NM_005282.1 | GPR4 | 2828 | 9.85 | -5.01 | 2.93 | -1.18 | 9.54E-04 |
| ILMN_21430 | NM_152611.2 | C20ORF75 | 164312 | 8.91 | -5.00 | 2.90 | -1.11 | 9.74E-04 |
| ILMN_21715 | NM_014398.2 | LAMP3 | 27074 | 13.12 | -5.00 | 2.90 | -1.61 | 9.74E-04 |
| ILMN_28694 | NM_001792.2 | CDH2 | 1000 | 9.21 | 4.99 | 2.86 | 1.45 | 1.00E-03 |
| ILMN_22283 | NM_003063.1 | SLN | 6588 | 9.93 | 4.98 | 2.84 | 2.08 | 1.01E-03 |
| ILMN_22857 | NM_001001437.3 | CCL3L3 | 414062 | 11.05 | 4.98 | 2.85 | 2.03 | 1.01E-03 |
| ILMN_10700 | NM_021965.3 | PGM5 | 5239 | 11.56 | 4.98 | 2.84 | 1.06 | 1.01E-03 |
| ILMN_25028 | NM_001063.2 | TF | 7018 | 8.40 | 4.98 | 2.83 | 1.00 | 1.02E-03 |
| ILMN_29396 | NM_005084.2 | PLA2G7 | 7941 | 10.57 | 4.94 | 2.72 | 1.56 | 1.11E-03 |
| ILMN_24855 | NM_021101.3 | CLDN1 | 9076 | 11.38 | 4.93 | 2.70 | 1.53 | 1.12E-03 |
| ILMN_9361 | NM_199127.1 | GGTL4 | 91227 | 8.36 | -4.91 | 2.63 | -1.02 | 1.18E-03 |
| ILMN_176190 | NM_001040114.1 | MYH11 | 4629 | 13.87 | 4.86 | 2.51 | 1.11 | 1.29E-03 |
| ILMN_21254 | NM_024788.2 | FLJ21062 | 79846 | 9.97 | 4.86 | 2.51 | 1.01 | 1.29E-03 |
| ILMN_14249 | NM_198148.1 | CPXM2 | 119587 | 10.44 | 4.83 | 2.43 | 1.41 | 1.37E-03 |
| ILMN_16399 | NM_006086.2 | TUBB3 | 10381 | 9.32 | 4.82 | 2.40 | 1.20 | 1.41E-03 |
| ILMN_3875 | NM_001078.2 | VCAM1 | 7412 | 11.48 | 4.81 | 2.37 | 2.18 | 1.44E-03 |
| ILMN_15660 | NM_014839.3 | LPPR4 | 9890 | 8.46 | 4.80 | 2.32 | 1.32 | 1.48E-03 |
| ILMN_179575 | NM_000584.2 | IL8 | 3576 | 12.40 | 4.78 | 2.27 | 1.78 | 1.54E-03 |
| ILMN_19972 | NM_020384.2 | CLDN2 | 9075 | 8.27 | 4.77 | 2.25 | 1.42 | 1.57E-03 |
| ILMN_2381 | NM_000804.2 | FOLR3 | 2352 | 8.52 | -4.77 | 2.24 | -1.89 | 1.57E-03 |
| ILMN_23060 | NM_000089.3 | COL1A2 | 1278 | 12.60 | 4.73 | 2.14 | 1.69 | 1.71E-03 |
| ILMN_9299 | NM_014392.2 | D4S234E | 27065 | 8.98 | 4.73 | 2.13 | 1.29 | 1.71E-03 |
| ILMN_27281 | NM_004887.3 | CXCL14 | 9547 | 9.17 | 4.72 | 2.12 | 1.37 | 1.73E-03 |
| ILMN_12987 | NM_000493.2 | COL10A1 | 1300 | 8.11 | 4.71 | 2.07 | 1.18 | 1.79E-03 |
| ILMN_30346 | NM_016140.2 | CGI-38 | 51673 | 9.90 | 4.70 | 2.04 | 1.39 | 1.84E-03 |
| ILMN_26242 | NM_019609.3 | CPXM | 56265 | 9.05 | 4.66 | 1.94 | 1.35 | 1.98E-03 |
| ILMN_3962 | NM_016509.2 | CLEC1B | 51266 | 7.53 | -4.66 | 1.94 | -1.33 | 1.98E-03 |
| ILMN_6158 | NM_021006.4 | CCL3L1 | 6349 | 9.37 | 4.64 | 1.88 | 1.61 | 2.06E-03 |
| ILMN_7811 | NM_205854.1 | SFTPG | 389376 | 12.53 | -4.62 | 1.82 | -1.38 | 2.15E-03 |
| ILMN_23783 | NM_198447.1 | GOLT1A | 127845 | 9.37 | -4.61 | 1.80 | -1.06 | 2.19E-03 |
| ILMN_16130 | NM_006997.2 | TACC2 | 10579 | 11.38 | -4.61 | 1.78 | -1.20 | 2.22E-03 |
| ILMN_11539 | NM_153283.1 | HYAL1 | 3373 | 10.57 | -4.60 | 1.76 | -1.02 | 2.25E-03 |
| ILMN_24839 | NM_138370.1 | LOC91461 | 91461 | 9.75 | -4.59 | 1.75 | -1.00 | 2.27E-03 |
| ILMN_18675 | NM_152717.1 | MGC35295 | 219995 | 8.25 | -4.57 | 1.68 | -1.27 | 2.41E-03 |
| ILMN_12248 | NM_001710.4 | CFB | 629 | 12.56 | 4.57 | 1.67 | 1.02 | 2.42E-03 |
| ILMN_20133 | NM_006398.2 | UBD | 10537 | 9.92 | 4.56 | 1.65 | 1.76 | 2.43E-03 |
| ILMN_26899 | NM_001071.1 | TYMS | 7298 | 9.30 | 4.55 | 1.63 | 1.08 | 2.47E-03 |
| ILMN_5478 | NM_006574.2 | CSPG5 | 10675 | 8.37 | -4.53 | 1.57 | -1.02 | 2.57E-03 |
| ILMN_11660 | NM_138969.2 | RDHE2 | 195814 | 9.73 | -4.53 | 1.56 | -1.28 | 2.60E-03 |
| ILMN_16214 | NM_005940.3 | MMP11 | 4320 | 8.99 | 4.51 | 1.53 | 2.00 | 2.65E-03 |
| ILMN_8467 | NM_173843.1 | IL1RN | 3557 | 9.79 | 4.49 | 1.46 | 1.40 | 2.78E-03 |
| ILMN_29392 | NM_001299.4 | CNN1 | 1264 | 10.94 | 4.48 | 1.42 | 1.39 | 2.87E-03 |
| ILMN_9434 | NM_003256.2 | TIMP4 | 7079 | 8.46 | -4.47 | 1.41 | -1.09 | 2.89E-03 |
| ILMN_19892 | NM_016413.3 | CPB2 | 1361 | 7.96 | -4.46 | 1.37 | -1.09 | 2.96E-03 |
| ILMN_14950 | NM_002251.3 | KCNS1 | 3787 | 7.95 | -4.45 | 1.33 | -1.05 | 3.05E-03 |
| ILMN_7706 | NM_017709.2 | FAM46C | 54855 | 11.83 | 4.44 | 1.30 | 1.16 | 3.10E-03 |
| ILMN_18883 | NM_001608.2 | ACADL | 33 | 9.01 | -4.41 | 1.22 | -1.34 | 3.30E-03 |
| ILMN_1999 | NM_002983.1 | CCL3 | 6348 | 10.10 | 4.39 | 1.18 | 1.67 | 3.40E-03 |
| ILMN_25431 | NM_005063.4 | SCD | 6319 | 13.11 | -4.38 | 1.15 | -1.10 | 3.49E-03 |
| ILMN_10476 | NM_022718.2 | MMP25 | 64386 | 8.52 | -4.34 | 1.03 | -1.02 | 3.83E-03 |
| ILMN_27932 | NM_006875.2 | PIM2 | 11040 | 11.31 | 4.33 | 1.01 | 1.24 | 3.90E-03 |
| ILMN_10372 | NM_006274.2 | CCL19 | 6363 | 10.30 | 4.32 | 0.98 | 1.95 | 3.98E-03 |
| ILMN_7069 | NM_001002915.1 | IGFL2 | 147920 | 7.90 | 4.31 | 0.96 | 1.17 | 4.04E-03 |
| ILMN_21799 | NM_001763.1 | CD1A | 909 | 8.86 | 4.30 | 0.94 | 1.48 | 4.09E-03 |
| ILMN_15617 | NM_003843.2 | SCEL | 8796 | 8.85 | -4.28 | 0.87 | -1.05 | 4.30E-03 |
| ILMN_18800 | NM_001089.1 | ABCA3 | 21 | 11.34 | -4.28 | 0.86 | -1.57 | 4.32E-03 |
| ILMN_26717 | NM_006308.1 | HSPB3 | 8988 | 9.00 | 4.27 | 0.85 | 1.21 | 4.33E-03 |
| ILMN_22396 | NM_002630.1 | PGC | 5225 | 14.13 | -4.27 | 0.86 | -1.36 | 4.33E-03 |
| ILMN_7523 | NM_001322.2 | CST2 | 1470 | 8.03 | 4.27 | 0.84 | 1.23 | 4.38E-03 |
| ILMN_17948 | NM_001927.3 | DES | 1674 | 10.65 | 4.25 | 0.79 | 1.54 | 4.54E-03 |
| ILMN_16682 | NM_005585.2 | SMAD6 | 4091 | 11.07 | -4.25 | 0.78 | -1.52 | 4.55E-03 |
| ILMN_16084 | NM_003853.2 | IL18RAP | 8807 | 10.10 | -4.24 | 0.77 | -1.14 | 4.61E-03 |
| ILMN_26004 | NM_213600.2 | PLA2G4F | 255189 | 10.11 | -4.24 | 0.77 | -1.58 | 4.61E-03 |
| ILMN_27029 | NM_004364.2 | CEBPA | 1050 | 11.15 | -4.22 | 0.71 | -1.06 | 4.82E-03 |
| ILMN_10629 | NM_000679.3 | ADRA1B | 147 | 8.64 | -4.22 | 0.70 | -1.02 | 4.85E-03 |
| ILMN_20280 | NM_145263.1 | SPATA18 | 132671 | 10.73 | 4.20 | 0.64 | 1.73 | 5.05E-03 |
| ILMN_13160 | NM_002639.2 | SERPINB5 | 5268 | 8.67 | 4.19 | 0.64 | 1.81 | 5.06E-03 |
| ILMN_7654 | NM_000424.2 | KRT5 | 3852 | 9.02 | 4.12 | 0.42 | 2.30 | 6.00E-03 |
| ILMN_26128 | NM_000090.2 | COL3A1 | 1281 | 12.94 | 4.11 | 0.40 | 1.83 | 6.12E-03 |
| ILMN_16493 | NM_005358.3 | LMO7 | 4008 | 9.67 | -4.10 | 0.38 | -1.21 | 6.17E-03 |
| ILMN_6390 | NM_000691.3 | ALDH3A1 | 218 | 9.85 | 4.07 | 0.29 | 1.94 | 6.59E-03 |
| ILMN_16434 | NM_005408.2 | CCL13 | 6357 | 11.11 | 4.06 | 0.28 | 1.01 | 6.62E-03 |
| ILMN_24564 | NM_001033087.1 | C20ORF133 | 140733 | 8.69 | -4.03 | 0.18 | -1.13 | 7.11E-03 |
| ILMN_30031 | NM_145740.2 | GSTA1 | 2938 | 10.08 | 4.01 | 0.14 | 2.22 | 7.38E-03 |
| ILMN_11123 | NM_018192.2 | LEPREL1 | 55214 | 11.67 | -4.00 | 0.12 | -1.17 | 7.46E-03 |
| ILMN_15989 | NM_001005505.1 | CACNA2D2 | 9254 | 10.27 | -4.00 | 0.11 | -1.29 | 7.47E-03 |
| ILMN_171409 | NM_003722.3 | TP73L | 8626 | 9.17 | 3.98 | 0.04 | 1.73 | 7.80E-03 |
| ILMN_1597 | NM_198452.1 | PNCK | 139728 | 8.64 | 3.96 | 0.01 | 1.23 | 7.96E-03 |
| ILMN_14571 | NM_032034.1 | SLC4A11 | 83959 | 9.44 | 3.95 | -0.02 | 1.46 | 8.17E-03 |
| ILMN_12288 | NM_020879.1 | KIAA1505 | 57639 | 9.97 | 3.94 | -0.06 | 1.44 | 8.35E-03 |
| ILMN_26343 | NM_004004.3 | GJB2 | 2706 | 8.25 | 3.92 | -0.10 | 1.07 | 8.58E-03 |
| ILMN_41107 | XM_939320.1 | LOC389816 | 389816 | 9.01 | 3.92 | -0.10 | 1.64 | 8.60E-03 |
| ILMN_2826 | NM_002234.2 | KCNA5 | 3741 | 9.57 | 3.89 | -0.18 | 1.03 | 9.22E-03 |
| ILMN_28510 | NM_025251.1 | KIAA1688 | 80728 | 9.49 | 3.88 | -0.21 | 1.16 | 9.33E-03 |
| ILMN_23424 | NM_005400.2 | PRKCE | 5581 | 10.00 | -3.88 | -0.21 | -1.02 | 9.33E-03 |
| ILMN_8057 | NM_003956.2 | CH25H | 9023 | 11.17 | 3.87 | -0.24 | 1.29 | 9.57E-03 |
| ILMN_30234 | NM_003856.2 | IL1RL1 | 9173 | 10.26 | -3.87 | -0.25 | -2.32 | 9.59E-03 |
| ILMN_28994 | NM_144565.2 | NIP | 90527 | 10.58 | -3.87 | -0.25 | -1.13 | 9.62E-03 |
| ILMN_14283 | NM_080617.4 | CBLN4 | 140689 | 8.29 | 3.85 | -0.29 | 1.30 | 9.91E-03 |
| ILMN_9752 | NM_006820.1 | IFI44L | 10964 | 10.53 | 3.85 | -0.30 | 1.18 | 1.00E-02 |
| ILMN_13072 | NM_002964.3 | S100A8 | 6279 | 13.09 | -3.83 | -0.35 | -1.31 | 1.03E-02 |
| ILMN_19350 | NM_178452.3 | LRRC50 | 123872 | 10.43 | 3.82 | -0.38 | 1.87 | 1.06E-02 |
| ILMN_137665 | NM_018414.2 | ST6GALNAC1 | 55808 | 9.22 | 3.80 | -0.43 | 1.28 | 1.10E-02 |
| ILMN_3636 | NM_003280.1 | TNNC1 | 7134 | 10.20 | -3.78 | -0.48 | -1.25 | 1.14E-02 |
| ILMN_2606 | NM_014326.3 | DAPK2 | 23604 | 9.30 | -3.78 | -0.49 | -1.01 | 1.15E-02 |
| ILMN_21913 | NM_017420.2 | SIX4 | 51804 | 9.46 | 3.75 | -0.57 | 1.25 | 1.22E-02 |
| ILMN_23751 | NM_182536.2 | GDDR | 200504 | 12.10 | -3.73 | -0.62 | -1.43 | 1.26E-02 |
| ILMN_23214 | NM_015225.1 | KIAA0367 | 23273 | 10.05 | 3.69 | -0.72 | 1.09 | 1.37E-02 |
| ILMN_27565 | NM_004624.2 | VIPR1 | 7433 | 12.60 | -3.67 | -0.77 | -1.55 | 1.42E-02 |
| ILMN_6391 | NM_001458.2 | FLNC | 2318 | 9.81 | 3.66 | -0.81 | 1.40 | 1.46E-02 |
| ILMN_6606 | NM_000717.2 | CA4 | 762 | 10.84 | -3.65 | -0.83 | -1.92 | 1.50E-02 |
| ILMN_7408 | NM_032411.1 | ECRG4 | 84417 | 10.73 | 3.64 | -0.86 | 1.43 | 1.52E-02 |
| ILMN_21252 | NM_015668.2 | RGS22 | 26166 | 9.01 | 3.63 | -0.87 | 1.15 | 1.53E-02 |
| ILMN_18011 | NM_152547.2 | BTNL9 | 153579 | 8.67 | -3.62 | -0.91 | -1.16 | 1.57E-02 |
| ILMN_25646 | NM_002965.2 | S100A9 | 6280 | 13.26 | -3.61 | -0.92 | -1.20 | 1.58E-02 |
| ILMN_183137 | NM_001042507.1 | LOC653499 | 653499 | 8.69 | 3.60 | -0.95 | 2.00 | 1.62E-02 |
| ILMN_15061 | NM_004237.2 | TRIP13 | 9319 | 9.24 | 3.60 | -0.96 | 1.27 | 1.63E-02 |
| ILMN_24314 | NM_004024.3 | ATF3 | 467 | 10.15 | 3.59 | -0.97 | 1.12 | 1.64E-02 |
| ILMN_10543 | NM_022068.1 | FAM38B | 63895 | 10.39 | 3.59 | -0.99 | 1.13 | 1.65E-02 |
| ILMN_7147 | NM_001393.2 | ECM2 | 1842 | 10.05 | 3.59 | -0.99 | 1.08 | 1.65E-02 |
| ILMN_4819 | NM_001001435.2 | CCL4L1 | 9560 | 8.19 | 3.58 | -1.00 | 1.23 | 1.67E-02 |
| ILMN_19978 | NM_006235.1 | POU2AF1 | 5450 | 8.83 | 3.58 | -1.01 | 1.18 | 1.68E-02 |
| ILMN_167992 | NM_003617.2 | RGS5 | 8490 | 9.06 | 3.57 | -1.03 | 1.11 | 1.69E-02 |
| ILMN_9681 | NM_006763.2 | BTG2 | 7832 | 12.60 | 3.55 | -1.07 | 1.17 | 1.74E-02 |
| ILMN_20406 | NM_004669.2 | CLIC3 | 9022 | 10.29 | -3.55 | -1.09 | -1.03 | 1.76E-02 |
| ILMN_9861 | NM_002112.1 | HDC | 3067 | 10.88 | 3.55 | -1.09 | 1.33 | 1.76E-02 |
| ILMN_23716 | NM_138461.2 | TM4SF19 | 116211 | 8.42 | 3.55 | -1.09 | 1.10 | 1.76E-02 |
| ILMN_27352 | NM_053277.1 | CLIC6 | 54102 | 12.41 | 3.54 | -1.12 | 1.28 | 1.81E-02 |
| ILMN_8954 | NM_001072.2 | UGT1A6 | 54578 | 8.08 | 3.52 | -1.16 | 1.13 | 1.86E-02 |
| ILMN_22039 | NM_000934.1 | SERPINF2 | 5345 | 9.90 | -3.51 | -1.18 | -1.01 | 1.89E-02 |
| ILMN_15389 | NM_007281.1 | SCRG1 | 11341 | 9.02 | 3.49 | -1.23 | 1.24 | 1.96E-02 |
| ILMN_137533 | NM_000393.2 | COL5A2 | 1290 | 11.72 | 3.49 | -1.23 | 1.08 | 1.96E-02 |
| ILMN_23377 | NM_033197.2 | C20ORF114 | 92747 | 13.08 | 3.48 | -1.25 | 3.45 | 1.99E-02 |
| ILMN_20817 | NM_021615.3 | CHST6 | 4166 | 8.73 | 3.48 | -1.27 | 1.27 | 2.01E-02 |
| ILMN_15135 | NM_001013675.1 | LOC400891 | 400891 | 8.35 | 3.47 | -1.30 | 1.22 | 2.05E-02 |
| ILMN_27783 | NM_001010846.1 | SHE | 126669 | 8.92 | -3.46 | -1.31 | -1.08 | 2.06E-02 |
| ILMN_11525 | NM_000041.2 | APOE | 348 | 14.08 | 3.44 | -1.37 | 1.09 | 2.15E-02 |
| ILMN_30351 | NM_002307.1 | LGALS7 | 3963 | 8.67 | 3.42 | -1.41 | 1.65 | 2.23E-02 |
| ILMN_29440 | NM_024626.1 | VTCN1 | 79679 | 8.41 | 3.42 | -1.42 | 1.44 | 2.25E-02 |
| ILMN_12592 | NM_206833.1 | CTXN1 | 404217 | 10.72 | 3.40 | -1.48 | 1.48 | 2.36E-02 |
| ILMN_8027 | NM_018365.1 | MNS1 | 55329 | 9.20 | 3.38 | -1.51 | 1.18 | 2.42E-02 |
| ILMN_17138 | NM_000607.1 | ORM1 | 5004 | 8.88 | -3.38 | -1.51 | -1.22 | 2.42E-02 |
| ILMN_26619 | NM_000055.1 | BCHE | 590 | 8.94 | 3.38 | -1.52 | 1.21 | 2.43E-02 |
| ILMN_16098 | NM_003247.2 | THBS2 | 7058 | 11.66 | 3.37 | -1.55 | 1.13 | 2.51E-02 |
| ILMN_9269 | NM_024889.3 | C10ORF81 | 79949 | 9.23 | 3.36 | -1.57 | 1.87 | 2.53E-02 |
| ILMN_23222 | NM_018602.2 | DNAJA4 | 55466 | 11.67 | 3.33 | -1.65 | 1.23 | 2.68E-02 |
| ILMN_23624 | NM_001453.1 | FOXC1 | 2296 | 11.72 | 3.32 | -1.67 | 1.04 | 2.71E-02 |
| ILMN_25604 | NM_144575.2 | CAPN13 | 92291 | 10.51 | 3.32 | -1.67 | 1.68 | 2.72E-02 |
| ILMN_11306 | NM_005411.3 | SFTPA1 | 6435 | 11.01 | -3.32 | -1.68 | -1.30 | 2.73E-02 |
| ILMN_25185 | NM_002982.3 | CCL2 | 6347 | 12.87 | 3.32 | -1.68 | 1.42 | 2.73E-02 |
| ILMN_13171 | NM_006419.1 | CXCL13 | 10563 | 8.10 | 3.31 | -1.68 | 1.06 | 2.75E-02 |
| ILMN_181020 | NM_003890.1 | FCGBP | 8857 | 10.74 | 3.30 | -1.71 | 1.19 | 2.79E-02 |
| ILMN_169048 | NM_006926.1 | SFTPA2 | 6436 | 12.14 | -3.30 | -1.72 | -1.75 | 2.82E-02 |
| ILMN_6469 | NM_000600.1 | IL6 | 3569 | 11.80 | 3.29 | -1.74 | 1.64 | 2.86E-02 |
| ILMN_16203 | NM_033285.2 | TP53INP1 | 94241 | 8.99 | 3.27 | -1.78 | 1.00 | 2.95E-02 |
| ILMN_6723 | NM_173452.1 | FCN3 | 8547 | 13.52 | -3.27 | -1.78 | -1.53 | 2.95E-02 |
| ILMN_12444 | NM_001023565.1 | LOC401286 | 401286 | 9.17 | -3.26 | -1.83 | -1.44 | 3.04E-02 |
| ILMN_4387 | NM_001008226.1 | DKFZP666G057 | 283726 | 7.87 | 3.25 | -1.84 | 1.00 | 3.06E-02 |
| ILMN_15634 | NM_005623.2 | CCL8 | 6355 | 10.42 | 3.25 | -1.85 | 1.22 | 3.10E-02 |
| ILMN_22279 | NM_015507.2 | EGFL6 | 25975 | 10.08 | 3.24 | -1.88 | 1.12 | 3.16E-02 |
| ILMN_3189 | NM_002275.2 | KRT15 | 3866 | 10.21 | 3.23 | -1.89 | 1.87 | 3.17E-02 |
| ILMN_2688 | NM_004864.1 | GDF15 | 9518 | 11.39 | 3.23 | -1.89 | 1.13 | 3.17E-02 |
| ILMN_4340 | NM_020070.2 | IGLL1 | 3543 | 13.31 | 3.23 | -1.90 | 1.25 | 3.20E-02 |
| ILMN_2146 | NM_000036.1 | AMPD1 | 270 | 8.05 | 3.22 | -1.92 | 1.02 | 3.24E-02 |
| ILMN_7052 | NM_005727.2 | TSPAN1 | 10103 | 10.18 | 3.21 | -1.94 | 1.60 | 3.30E-02 |
| ILMN_26383 | NM_006159.1 | NELL2 | 4753 | 9.39 | 3.17 | -2.04 | 1.28 | 3.53E-02 |
| ILMN_16264 | NM_031422.1 | CHST9 | 83539 | 9.09 | 3.16 | -2.07 | 1.61 | 3.63E-02 |
| ILMN_8636 | NM_016459.3 | MGC29506 | 51237 | 11.16 | 3.16 | -2.08 | 1.50 | 3.63E-02 |
| ILMN_4881 | NM_018286.1 | TMEM100 | 55273 | 11.35 | -3.14 | -2.11 | -1.53 | 3.72E-02 |
| ILMN_16401 | NM_018272.2 | CASC1 | 55259 | 9.53 | 3.11 | -2.18 | 1.44 | 3.92E-02 |
| ILMN_18560 | NM_152676.1 | FBXO15 | 201456 | 9.61 | 3.11 | -2.18 | 1.49 | 3.92E-02 |
| ILMN_22732 | NM_002985.2 | CCL5 | 6352 | 12.12 | 3.10 | -2.21 | 1.05 | 4.03E-02 |
| ILMN_11325 | NM_003914.2 | CCNA1 | 8900 | 8.69 | 3.10 | -2.21 | 1.09 | 4.03E-02 |
| ILMN_4012 | NM_000352.2 | ABCC8 | 6833 | 8.49 | -3.07 | -2.29 | -1.05 | 4.26E-02 |
| ILMN_167544 | NM_001013618.1 | LOC91353 | 91353 | 9.03 | 3.06 | -2.31 | 1.57 | 4.31E-02 |
| ILMN_9648 | NM_017425.2 | SPA17 | 53340 | 10.43 | 3.04 | -2.35 | 1.11 | 4.44E-02 |
| ILMN_176256 | NM_004895.3 | NLRP3 | 114548 | 9.81 | 2.99 | -2.47 | 1.07 | 4.89E-02 |
| ILMN_27842 | NM_145286.1 | STOML3 | 161003 | 8.22 | 2.99 | -2.47 | 1.05 | 4.89E-02 |
| ILMN_25950 | NM_014767.1 | SPOCK2 | 9806 | 12.34 | -2.98 | -2.49 | -1.08 | 4.98E-02 |
| ILMN_26267 | NM_000065.1 | C6 | 729 | 10.30 | 2.97 | -2.52 | 1.42 | 5.07E-02 |
| ILMN_13594 | NM_003551.2 | NME5 | 8382 | 9.15 | 2.96 | -2.53 | 1.02 | 5.14E-02 |
| ILMN_21335 | NM_001838.2 | CCR7 | 1236 | 8.81 | 2.96 | -2.54 | 1.12 | 5.18E-02 |
| ILMN_11610 | NM_138770.1 | CCDC74A | 90557 | 8.78 | 2.95 | -2.57 | 1.06 | 5.26E-02 |
| ILMN_19037 | NM_152701.2 | ABCA13 | 154664 | 8.08 | 2.95 | -2.58 | 1.08 | 5.29E-02 |
| ILMN_8623 | NM_007021.2 | C10ORF10 | 11067 | 12.78 | 2.94 | -2.60 | 1.40 | 5.38E-02 |
| ILMN_29397 | NM_178499.2 | CCDC60 | 160777 | 8.95 | 2.92 | -2.63 | 1.28 | 5.50E-02 |
| ILMN_3183 | NM_016354.3 | SLCO4A1 | 28231 | 10.31 | -2.91 | -2.66 | -1.13 | 5.63E-02 |
| ILMN_21263 | NM_030817.1 | APOLD1 | 81575 | 11.84 | 2.90 | -2.68 | 1.14 | 5.69E-02 |
| ILMN_25995 | NM_004633.3 | IL1R2 | 7850 | 9.13 | -2.89 | -2.71 | -1.10 | 5.76E-02 |
| ILMN_14698 | NM_182508.1 | FLJ40919 | 144809 | 10.28 | 2.89 | -2.71 | 1.74 | 5.78E-02 |
| ILMN_3644 | NM_018897.1 | DNAH7 | 56171 | 8.79 | 2.86 | -2.78 | 1.06 | 6.10E-02 |
| ILMN_168618 | NM_007036.3 | ESM1 | 11082 | 8.73 | -2.84 | -2.83 | -1.37 | 6.30E-02 |
| ILMN_5066 | NM_001565.1 | CXCL10 | 3627 | 11.16 | 2.84 | -2.83 | 2.21 | 6.34E-02 |
| ILMN_24419 | NM_025145.4 | C10ORF79 | 80217 | 8.71 | 2.82 | -2.86 | 1.19 | 6.49E-02 |
| ILMN_19839 | NM_198469.1 | C9ORF18 | 254956 | 8.35 | 2.81 | -2.89 | 1.17 | 6.60E-02 |
| ILMN_138711 | NM_024687.1 | FLJ23049 | 79740 | 9.42 | 2.81 | -2.90 | 1.36 | 6.63E-02 |
| ILMN_14456 | NM_000185.3 | SERPIND1 | 3053 | 8.47 | 2.81 | -2.90 | 1.20 | 6.65E-02 |
| ILMN_15794 | NM_033413.2 | LRRC46 | 90506 | 8.80 | 2.80 | -2.91 | 1.14 | 6.67E-02 |
| ILMN_4791 | NM_144647.2 | CAPSL | 133690 | 8.60 | 2.80 | -2.91 | 1.16 | 6.69E-02 |
| ILMN_21827 | NM_006017.1 | PROM1 | 8842 | 10.85 | 2.80 | -2.92 | 1.92 | 6.70E-02 |
| ILMN_37677 | NM_021601.3 | CD79A | 973 | 8.98 | 2.78 | -2.96 | 1.11 | 6.89E-02 |
| ILMN_20473 | NM_005554.3 | KRT6A | 3853 | 9.64 | 2.78 | -2.96 | 2.15 | 6.90E-02 |
| ILMN_7438 | NM_003645.2 | SLC27A2 | 11001 | 9.38 | 2.78 | -2.96 | 1.07 | 6.91E-02 |
| ILMN_9579 | NM_152500.1 | CCDC17 | 149483 | 9.72 | 2.78 | -2.97 | 1.44 | 6.98E-02 |
| ILMN_20794 | NM_005564.2 | LCN2 | 3934 | 12.51 | 2.76 | -3.00 | 1.51 | 7.11E-02 |
| ILMN_9005 | NM_182520.1 | C22ORF15 | 150248 | 9.52 | 2.76 | -3.00 | 1.44 | 7.15E-02 |
| ILMN_20631 | NM_032930.1 | C11ORF70 | 85016 | 9.52 | 2.74 | -3.04 | 1.17 | 7.33E-02 |
| ILMN_23096 | NM_052863.2 | SCGB3A1 | 92304 | 14.54 | 2.72 | -3.09 | 1.56 | 7.57E-02 |
| ILMN_13178 | NM_020877.2 | DNHD3 | 146754 | 9.76 | 2.72 | -3.09 | 1.39 | 7.57E-02 |
| ILMN_18215 | NM_178456.2 | C20ORF85 | 128602 | 10.34 | 2.72 | -3.10 | 1.84 | 7.63E-02 |
| ILMN_19771 | NM_138634.1 | MSMB | 4477 | 8.59 | 2.71 | -3.11 | 1.65 | 7.66E-02 |
| ILMN_16523 | NM_144577.1 | FLJ32926 | 93233 | 8.40 | 2.71 | -3.13 | 1.07 | 7.75E-02 |
| ILMN_21973 | NM_004058.2 | CAPS | 828 | 10.91 | 2.70 | -3.13 | 1.56 | 7.77E-02 |
| ILMN_29244 | NM_025019.1 | TUBA4 | 80086 | 8.58 | 2.70 | -3.15 | 1.28 | 7.86E-02 |
| ILMN_28867 | NM_033364.2 | C3ORF15 | 89876 | 10.18 | 2.70 | -3.15 | 1.02 | 7.86E-02 |
| ILMN_4287 | NM_144668.3 | WDR66 | 144406 | 9.00 | 2.68 | -3.17 | 1.22 | 8.03E-02 |
| ILMN_9895 | NM_130897.1 | DYNLRB2 | 83657 | 10.44 | 2.68 | -3.18 | 1.48 | 8.04E-02 |
| ILMN_1949 | NM_024690.2 | MUC16 | 94025 | 9.31 | 2.68 | -3.18 | 1.70 | 8.05E-02 |
| ILMN_11864 | NM_001031743.1 | C6ORF165 | 154313 | 8.61 | 2.67 | -3.20 | 1.03 | 8.12E-02 |
| ILMN_35502 | XM_940314.1 | NLF2 | 388125 | 9.98 | 2.67 | -3.21 | 1.37 | 8.17E-02 |
| ILMN_165501 | NM_000774.3 | CYP2F1 | 1572 | 9.10 | 2.66 | -3.24 | 1.63 | 8.33E-02 |
| ILMN_26176 | NM_021870.2 | FGG | 2266 | 8.68 | -2.66 | -3.24 | -1.47 | 8.33E-02 |
| ILMN_3099 | NM_024593.2 | EFCAB1 | 79645 | 10.34 | 2.65 | -3.24 | 1.58 | 8.35E-02 |
| ILMN_28911 | NM_053285.1 | TEKT1 | 83659 | 9.97 | 2.65 | -3.25 | 1.56 | 8.39E-02 |
| ILMN_13603 | NM_006732.1 | FOSB | 2354 | 14.02 | 2.65 | -3.25 | 1.55 | 8.39E-02 |
| ILMN_19608 | NM_001002026.2 | CLDN18 | 51208 | 11.91 | -2.64 | -3.26 | -1.47 | 8.45E-02 |
| ILMN_2231 | NM_152750.2 | FLJ23834 | 222256 | 10.73 | 2.64 | -3.27 | 1.65 | 8.46E-02 |
| ILMN_23986 | NM_015896.2 | ZMYND10 | 51364 | 9.36 | 2.64 | -3.28 | 1.30 | 8.53E-02 |
| ILMN_2210 | NM_207430.1 | FLJ46266 | 399949 | 9.55 | 2.63 | -3.28 | 1.54 | 8.57E-02 |
| ILMN_14828 | NM_031421.1 | TTC25 | 83538 | 9.59 | 2.62 | -3.32 | 1.17 | 8.78E-02 |
| ILMN_14238 | NM_001010940.1 | LOC138255 | 138255 | 9.58 | 2.61 | -3.33 | 1.50 | 8.84E-02 |
| ILMN_6160 | NM_178504.3 | DNHD2 | 201625 | 10.02 | 2.59 | -3.37 | 1.60 | 9.10E-02 |
| ILMN_138843 | NM_001002912.2 | C1ORF173 | 127254 | 9.47 | 2.59 | -3.39 | 1.44 | 9.21E-02 |
| ILMN_8796 | NM_031294.2 | LRRC48 | 83450 | 8.98 | 2.58 | -3.39 | 1.09 | 9.22E-02 |
| ILMN_25383 | NM_207417.1 | FLJ46082 | 389799 | 8.77 | 2.56 | -3.44 | 1.29 | 9.53E-02 |
| ILMN_25691 | NM_152290.1 | C1ORF158 | 93190 | 8.64 | 2.55 | -3.48 | 1.09 | 9.75E-02 |
| ILMN_9635 | NM_172242.1 | SPAG6 | 9576 | 9.46 | 2.54 | -3.48 | 1.41 | 9.80E-02 |
| ILMN_15139 | NM_000846.3 | GSTA2 | 2939 | 9.19 | 2.54 | -3.49 | 1.58 | 9.81E-02 |
| ILMN_6194 | NM_144654.1 | C9ORF116 | 138162 | 10.14 | 2.54 | -3.49 | 1.38 | 9.87E-02 |
