# Supplementary material for: Prominence of IL6, IGF, TLR, and Bioenergetics Pathway Perturbation in Lung Tissues of Scleroderma Patients With Pulmonary Fibrosis
Source: Front Immunol. 2020 Mar 10;11:383. doi: 10.3389/fimmu.2020.00383 (PMC7075854; doi:10.3389/fimmu.2020.00383)
Supplement: Supplementary file 3 [file Table_3.DOCX]

***Supplementary Table 3***

Prominence of IL6, IGF, TLR and bioenergetics pathway perturbation in lung tissues of scleroderma patients with pulmonary fibrosis

**Ludivine Renaud****^1^, Willian A. da Silveira^2^, Naoko Takamura^1^, Gary Hardiman^2^, Carol Feghali-Bostwick^1^***

^1^ Department of Medicine, Medical University of South Carolina, Charleston, SC, USA.

^2^ School of Biological Sciences and Institute for Global Food Security, Queens University Belfast, Belfast BT9 5AG, UK.

*** Correspondence:**Dr. Carol Feghali-Bostwick
feghalib@musc.edu

**Supplementary Table 3: Gene expression profile of the intersection hub DE genes.** Expression values of the hug genes identified in the intersection between SSc-PF versus NL and IPF versus NL DE lists (q<0.1). Red: upregulated (log2FC>1), blue: downregulated (log2FC<-1). Sorted on q-value.

| SSc-PF versus NL | | | |  | IPF versus NL | | | |
| --- | --- | --- | --- | --- | --- | --- | --- | --- |
| HUGO_Symbol | Entrez_ID | log2FC | q-value |  | HUGO_Symbol | Entrez_ID | log2FC | q-value |
| COL9A2 | 1298 | 1.12 | 1.91E-05 |  | COL9A2 | 1298 | 1.10 | 2.60E-05 |
| COL7A1 | 1294 | 2.14 | 3.20E-05 |  | COL1A1 | 1277 | 2.29 | 4.71E-05 |
| COL15A1 | 1306 | 1.96 | 5.14E-05 |  | COL17A1 | 1308 | 1.94 | 1.31E-04 |
| COL17A1 | 1308 | 2.06 | 5.21E-05 |  | COL7A1 | 1294 | 1.92 | 1.56E-04 |
| SPP1 | 6696 | 3.67 | 1.35E-04 |  | CXCL12 | 6387 | 1.20 | 2.00E-04 |
| COL1A1 | 1277 | 2.09 | 1.42E-04 |  | COL15A1 | 1306 | 1.59 | 6.54E-04 |
| CDH2 | 1000 | 1.53 | 5.17E-04 |  | SPP1 | 6696 | 3.18 | 7.35E-04 |
| COL10A1 | 1300 | 1.31 | 5.53E-04 |  | CDH2 | 1000 | 1.45 | 1.00E-03 |
| VCAM1 | 7412 | 2.36 | 5.88E-04 |  | VCAM1 | 7412 | 2.18 | 1.44E-03 |
| CXCL12 | 6387 | 1.03 | 1.01E-03 |  | COL1A2 | 1278 | 1.69 | 1.71E-03 |
| COL1A2 | 1278 | 1.68 | 1.63E-03 |  | COL10A1 | 1300 | 1.18 | 1.79E-03 |
| COL3A1 | 1281 | 1.84 | 5.43E-03 |  | CCL19 | 6363 | 1.95 | 3.98E-03 |
| CCL2 | 6347 | 1.67 | 8.55E-03 |  | COL3A1 | 1281 | 1.83 | 6.12E-03 |
| CCL19 | 6363 | 1.71 | 1.06E-02 |  | COL5A2 | 1290 | 1.08 | 1.96E-02 |
| COL5A2 | 1290 | 1.13 | 1.46E-02 |  | CCL2 | 6347 | 1.42 | 2.73E-02 |
| SERPIND1 | 3053 | 1.40 | 2.85E-02 |  | IL6 | 3569 | 1.64 | 2.86E-02 |
| IL6 | 3569 | 1.61 | 3.05E-02 |  | CXCL10 | 3627 | 2.21 | 6.34E-02 |
| FGG | 2266 | -1.50 | 7.66E-02 |  | SERPIND1 | 3053 | 1.20 | 6.65E-02 |
| CXCL10 | 3627 | 2.02 | 9.43E-02 |  | FGG | 2266 | -1.47 | 8.33E-02 |
