# Supplementary material for: Prominence of IL6, IGF, TLR, and Bioenergetics Pathway Perturbation in Lung Tissues of Scleroderma Patients With Pulmonary Fibrosis
Source: Front Immunol. 2020 Mar 10;11:383. doi: 10.3389/fimmu.2020.00383 (PMC7075854; doi:10.3389/fimmu.2020.00383)
Supplement: Supplementary file 4 [file Table_4.docx]

***Supplementary Table 4***

Prominence of IL6, IGF, TLR and bioenergetics pathway perturbation in lung tissues of scleroderma patients with pulmonary fibrosis

**Ludivine Renaud****^1^, Willian A. da Silveira^2^, Naoko Takamura^1^, Gary Hardiman^2^, Carol Feghali-Bostwick^1^***

^1^ Department of Medicine, Medical University of South Carolina, Charleston, SC, USA.

^2^ School of Biological Sciences and Institute for Global Food Security, Queens University Belfast, Belfast BT9 5AG, UK.

*** Correspondence:**Dr. Carol Feghali-Bostwick
feghalib@musc.edu

**Supplementary Table 4: Gene expression profile of the hub DE genes unique to SSc-PF.** Expression values of the hug genes identified in the DE list unique to SSc-PF (q<0.1). Red: upregulated (log2FC>1), blue: downregulated (log2FC<-1). Sorted on q-value.

| HUGO_Symbol | Entrez_ID | log2FC | q-value |
| --- | --- | --- | --- |
| ARG1 | 383 | -1.03 | 1.38E-04 |
| TLR8 | 51311 | -1.10 | 8.32E-04 |
| MPO | 4353 | -1.01 | 2.75E-03 |
| PTGS2 | 5743 | 1.30 | 9.49E-03 |
