# Supplementary material for: Prominence of IL6, IGF, TLR, and Bioenergetics Pathway Perturbation in Lung Tissues of Scleroderma Patients With Pulmonary Fibrosis
Source: Front Immunol. 2020 Mar 10;11:383. doi: 10.3389/fimmu.2020.00383 (PMC7075854; doi:10.3389/fimmu.2020.00383)
Supplement: Supplementary file 5 [file Table_5.docx]

***Supplementary Table 5***

Prominence of IL6, IGF, TLR and bioenergetics pathway perturbation in lung tissues of scleroderma patients with pulmonary fibrosis

**Ludivine Renaud****^1^, Willian A. da Silveira^2^, Naoko Takamura^1^, Gary Hardiman^2^, Carol Feghali-Bostwick^1^***

^1^ Department of Medicine, Medical University of South Carolina, Charleston, SC, USA.

^2^ School of Biological Sciences and Institute for Global Food Security, Queens University Belfast, Belfast BT9 5AG, UK.

*** Correspondence:**Dr. Carol Feghali-Bostwick
feghalib@musc.edu

**Supplementary Table 5: Gene expression profile of the hub DE genes unique to IPF.** Expression values of the hug genes identified in the DE list unique to IPF (q<0.1). Red: upregulated (log2FC>1), blue: downregulated (log2FC<-1). Sorted on q-value.

| HUGO_Symbol | Entrez_ID | log2FC | q-value |
| --- | --- | --- | --- |
| CSF2 | 1437 | -1.20 | 1.15E-05 |
| CCL3 | 6348 | 1.67 | 3.40E-03 |
| CCL13 | 6357 | 1.01 | 6.62E-03 |
| CCL4L1 | 9560 | 1.23 | 1.67E-02 |
| APOE | 348 | 1.09 | 2.15E-02 |
| CXCL13 | 10563 | 1.06 | 2.75E-02 |
| CCL5 | 6352 | 1.05 | 4.03E-02 |
| NLRP3 | 114548 | 1.07 | 4.89E-02 |
| CCR7 | 1236 | 1.12 | 5.18E-02 |
