# Supplementary figures and images for: Prominence of IL6, IGF, TLR, and Bioenergetics Pathway Perturbation in Lung Tissues of Scleroderma Patients With Pulmonary Fibrosis
Source: Front Immunol. 2020 Mar 10;11:383. doi: 10.3389/fimmu.2020.00383 (PMC7075854; doi:10.3389/fimmu.2020.00383)

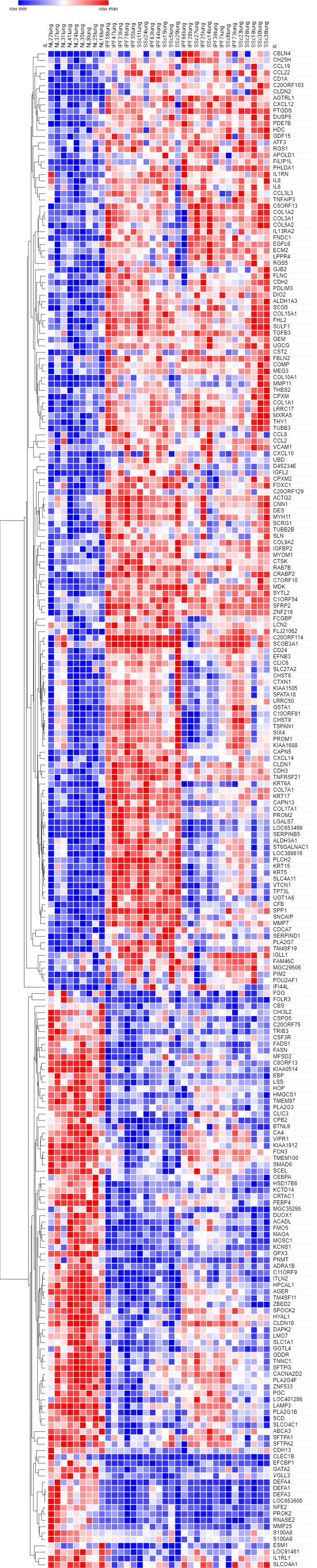

Supplement: Supplementary file 6 [file Image_1.png]
