# Supplementary material for: Prominence of IL6, IGF, TLR, and Bioenergetics Pathway Perturbation in Lung Tissues of Scleroderma Patients With Pulmonary Fibrosis
Source: Front Immunol. 2020 Mar 10;11:383. doi: 10.3389/fimmu.2020.00383 (PMC7075854; doi:10.3389/fimmu.2020.00383)
Supplement: Supplementary file 8 [file Image_3.PDF]

Supplementary Figure 3

Prominence of IL6, IGF, TLR and bioenergetics pathway perturbation in lung tissues of scleroderma patients with pulmonary fibrosis

Ludivine Renaud[1], Willian A. da Silveira[2], Naoko Takamura[1], Gary Hardiman[2], Carol Feghali-Bostwick[1]*

[1] Department of Medicine, Medical University of South Carolina, Charleston, SC, USA.

[2] School of Biological Sciences and Institute for Global Food Security, Queens University Belfast, Belfast BT9 5AG, UK.

*** Correspondence:**
Dr. Carol Feghali-Bostwick
feghalib@musc.edu

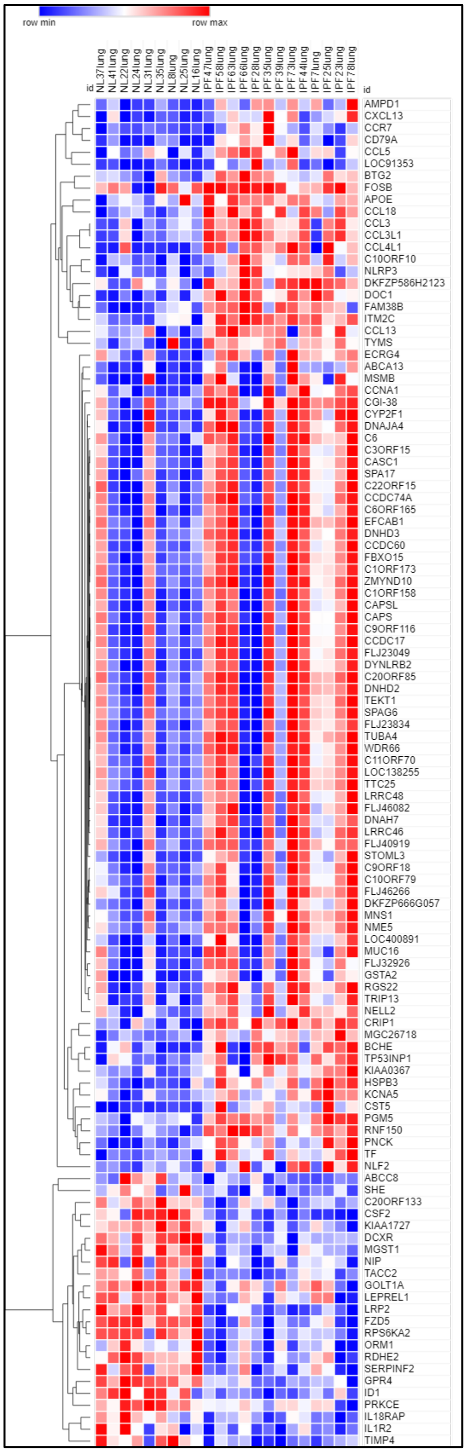

Supplementary Figure 3: Heatmap of DE genes unique to IPF.
