# Supplementary material for: Prominence of IL6, IGF, TLR, and Bioenergetics Pathway Perturbation in Lung Tissues of Scleroderma Patients With Pulmonary Fibrosis
Source: Front Immunol. 2020 Mar 10;11:383. doi: 10.3389/fimmu.2020.00383 (PMC7075854; doi:10.3389/fimmu.2020.00383)
Supplement: Supplementary file 11 [file Image_6.PDF]

Supplementary Figure 6

Prominence of IL6, IGF, TLR and bioenergetics pathway perturbation in lung tissues of scleroderma patients with pulmonary fibrosis

Ludivine Renaud[1], Willian A. da Silveira[2], Naoko Takamura[1], Gary Hardiman[2], Carol Feghali-Bostwick[1]*

[1] Department of Medicine, Medical University of South Carolina, Charleston, SC, USA.

[2] School of Biological Sciences and Institute for Global Food Security, Queens University Belfast, Belfast BT9 5AG, UK.

*** Correspondence:**
Dr. Carol Feghali-Bostwick
feghalib@musc.edu

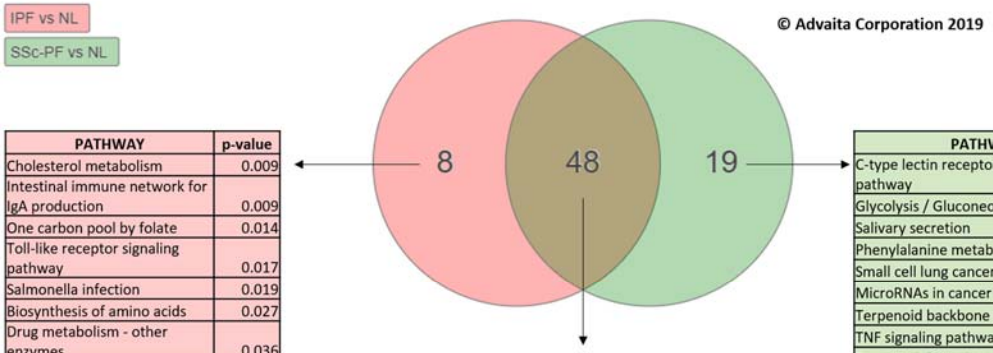

PATHWAY	p-value
Cholesterol metabolism	0.009
Intestinal immune network for IgA production	0.009
One carbon pool by folate	0.014
Toll-like receptor signaling pathway	0.017
Salmonella infection	0.019
Biosynthesis of amino acids	0.027
Drug metabolism - other enzymes	0.036
Hippo signaling pathway	0.040

PATHWAY	p-value
C-type lectin receptor signaling pathway	0.004
Glycolysis / Gluconeogenesis	0.006
Salivary secretion	0.006
Phenylalanine metabolism	0.009
Small cell lung cancer	0.009
MicroRNAs in cancer	0.014
Terpenoid backbone biosynthesis	0.019
TNF signaling pathway	0.022
Fatty acid degradation	0.022
Thyroid hormone signaling pathway	0.023
Breast cancer	0.024
Ether lipid metabolism	0.024
PI3K-Akt signaling pathway	0.026
PPAR signaling pathway	0.030
Signaling pathways regulating pluripotency of stem cells	0.037
AMPK signaling pathway	0.039
Melanogenesis	0.041
Biosynthesis of unsaturated fatty acids	0.044
Mucin type O-glycan biosynthesis	0.049

	IPF vs. NL	SSc-PF vs. NL
PATHWAY	p-value	p-value
Protein digestion and absorption	5.62E-08	2.00E-10
ECM-receptor interaction	3.20E-08	1.12E-08
Drug metabolism - cytochrome P450	1.68E-07	1.41E-07
Steroid biosynthesis	1.19E-06	1.08E-06
Cytokine-cytokine receptor interaction	8.18E-08	6.34E-06
Amoebiasis	0.0007	9.18E-06
Staphylococcus aureus infection	0.0058	1.95E-05
Arachidonic acid metabolism	0.0025	2.48E-05
Chemical carcinogenesis	0.0001	2.94E-05
Metabolic pathways	0.0035	3.14E-05
Chemokine signaling pathway	0.0000	3.43E-05
Malaria	0.0002	3.67E-05
Metabolism of xenobiotics by cytochrome P450	0.0001	4.52E-05
Tyrosine metabolism	0.0001	0.0001
Focal adhesion	0.0015	0.0001
Pathways in cancer	0.0057	0.0002
Human papillomavirus infection	0.0000	0.0006
Complement and coagulation cascades	0.0104	0.0008
Leukocyte transendothelial migration	0.0104	0.0014
AGE-RAGE signaling pathway in diabetic complications	0.0010	0.0015
Vascular smooth muscle contraction	0.0028	0.0015
Rheumatoid arthritis	0.0003	0.0026
Transcriptional misregulation in cancer	0.0053	0.0028
Histidine metabolism	0.0198	0.0033
Wnt signaling pathway	0.0065	0.0044
Dilated cardiomyopathy (DCM)	0.0111	0.0044
Glutathione metabolism	0.0050	0.0046
alpha-Linolenic acid metabolism	0.0271	0.0050
IL-17 signaling pathway	0.0092	0.0056
Chagas disease (American trypanosomiasis)	0.0251	0.0060
Hypertrophic cardiomyopathy (HCM)	0.0010	0.0085
Cell adhesion molecules (CAMs)	0.0001	0.0089
Basal cell carcinoma	0.0169	0.0091
Linoleic acid metabolism	0.0458	0.0101
NF-kappa B signaling pathway	0.0124	0.0121
Glycine, serine and threonine metabolism	0.0006	0.0124
Retinol metabolism	0.0140	0.0131
Valine, leucine and isoleucine biosynthesis	0.0144	0.0141
Antifolate resistance	0.0007	0.0141
Phagosome	0.0334	0.0143
Arrhythmogenic right ventricular cardiomyopathy	0.0022	0.0149
Taurine and hypotaurine metabolism	0.0159	0.0153
Acute myeloid leukemia	0.0460	0.0175
Hematopoietic cell lineage	0.0009	0.0193
Hepatocellular carcinoma	0.0240	0.0215
Cysteine and methionine metabolism	0.0254	0.0240
Platinum drug resistance	0.0188	0.0337
Selenocompound metabolism	0.0446	0.0432

Supplementary Figure 6: Meta-analysis of perturbed biological pathways in IPF and SSc-PF. Systems level analysis revealed 56 enriched pathways in the IPF versus NL comparison, and 67 in the SSc-PF versus NL ($q<0.1$, linear FC 1.5). 48 pathways were commonly enriched in both diseases (intersection). 8 and 19 pathways were exclusively perturbed in IPF and SSc-PF, respectively.
