# Supplementary material for: Prominence of IL6, IGF, TLR, and Bioenergetics Pathway Perturbation in Lung Tissues of Scleroderma Patients With Pulmonary Fibrosis
Source: Front Immunol. 2020 Mar 10;11:383. doi: 10.3389/fimmu.2020.00383 (PMC7075854; doi:10.3389/fimmu.2020.00383)
Supplement: Supplementary file 16 [file Image_11.PDF]

Supplementary Figure 11

Prominence of IL6, IGF, TLR and bioenergetics pathway perturbation in lung tissues of scleroderma patients with pulmonary fibrosis

Ludivine Renaud[1], Willian A. da Silveira[2], Naoko Takamura[1], Gary Hardiman[2], Carol Feghali-Bostwick[1]*

[1] Department of Medicine, Medical University of South Carolina, Charleston, SC, USA.

[2] School of Biological Sciences and Institute for Global Food Security, Queens University Belfast, Belfast BT9 5AG, UK.

*** Correspondence:**
Dr. Carol Feghali-Bostwick
feghalib@musc.edu

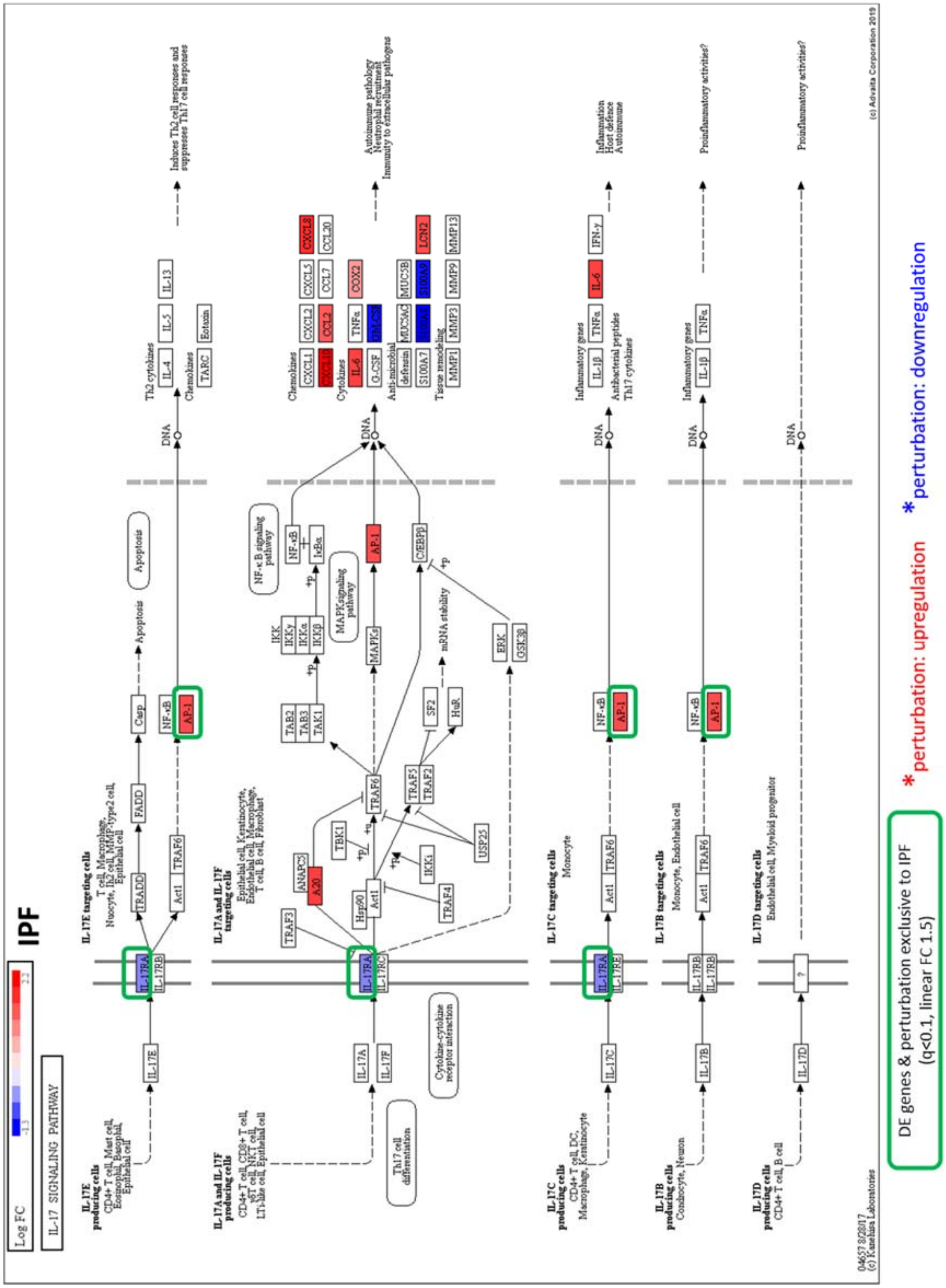

Supplementary Figure 11: IPF "IL-17 signaling pathway". This Advaita-generated figure shows DE genes (q<0.1) in the KEGG format. Red: upregulation (log2FC>0.6, linear FC increase of 1.5) and blue: downregulation (log2FC<-0.6, linear FC decrease of 1.5). Genes and perturbation of the pathway that are specific to IPF were added on in green according to Advaita impact analysis. Perturbation is shown by red and blue stars.
